# Supplementary material for: Global Protected Areas as refuges for amphibians and reptiles under climate change
Source: Nat Commun. 2023 Mar 13;14:1389. doi: 10.1038/s41467-023-36987-y (PMC10011414; doi:10.1038/s41467-023-36987-y)
Supplement: Supplementary file 1 — Supplementary Information [file 41467_2023_36987_MOESM1_ESM.pdf]

Supplementary Figures

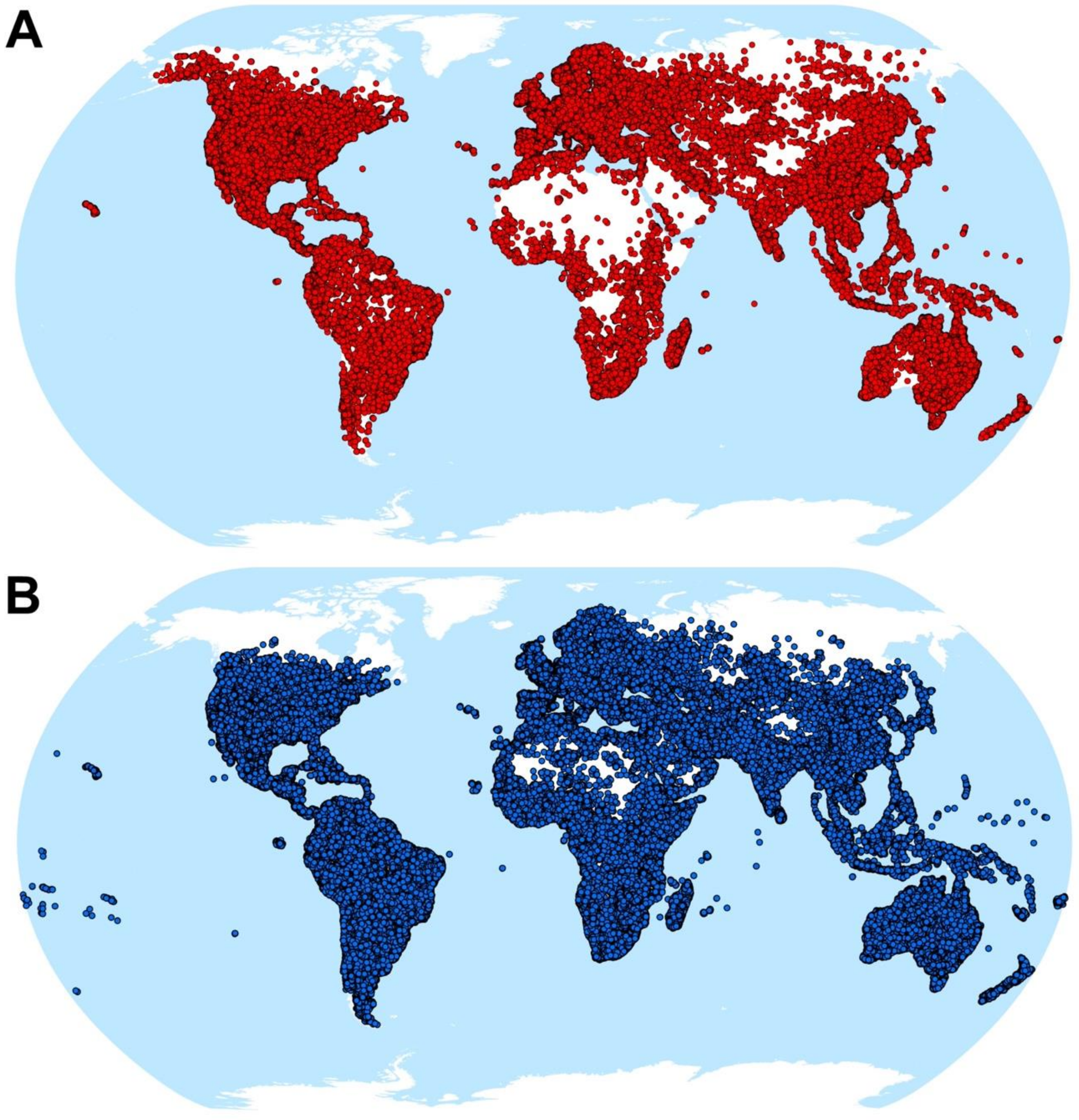

Supplementary Figure 1 Occurrence records of amphibians and reptiles. (A) Amphibians; (B) Reptiles
.

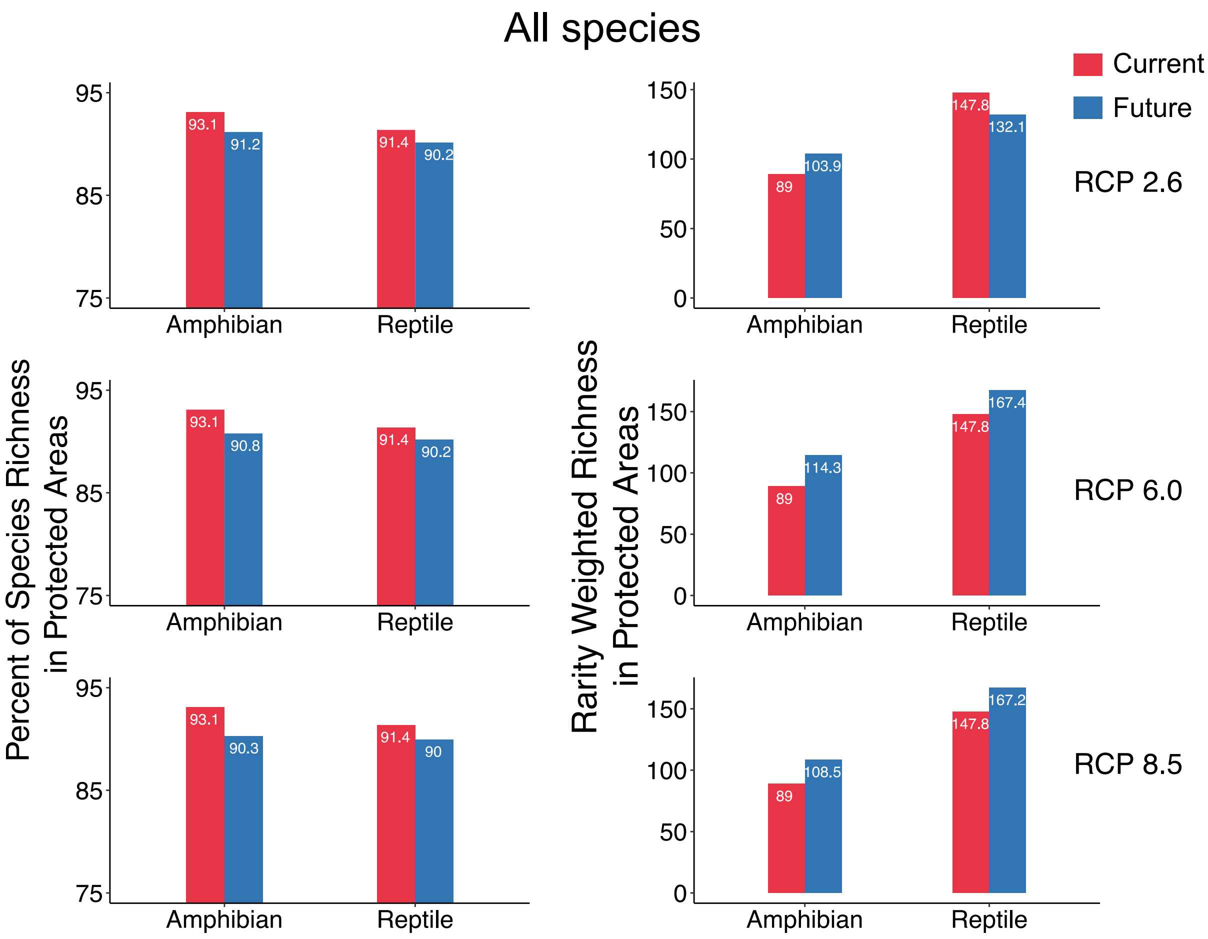

Supplementary Figure 2 Percent of species and rarity weighted richness in protected areas (PAs) at present and future (RCP 2.6, 6.0 and 8.5) by 2070. The first column represents the percent of species in PAs, the second column represents rarity weighted richness in PAs. The first to third rows represent RCP 2.6, 6.0 and 8.5. The red color indicates current, blue indicates by 2070. We assume future land use remains unchanged for this study.

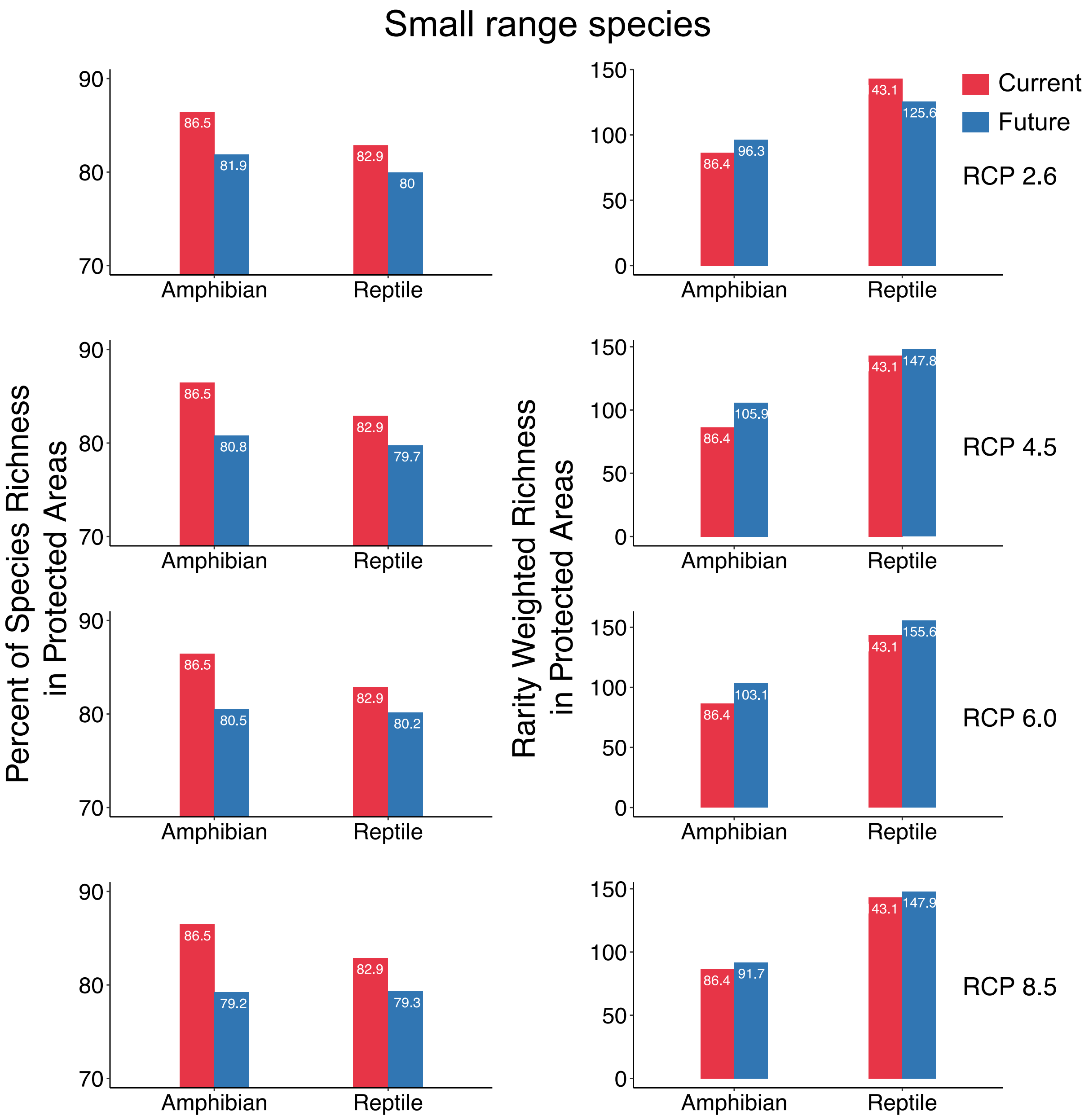

Supplementary Figure 3 Percent of small range species and their rarity weighted richness in protected areas (PAs). The first column represents the percent of species in PAs, the second column represents rarity weighted richness in PAs. The first to fourth rows represent RCP 2.6, 4.5, 6.0 and 8.5. The red color indicates current, blue indicates by 2070. We assume future land use remains unchanged for this study.

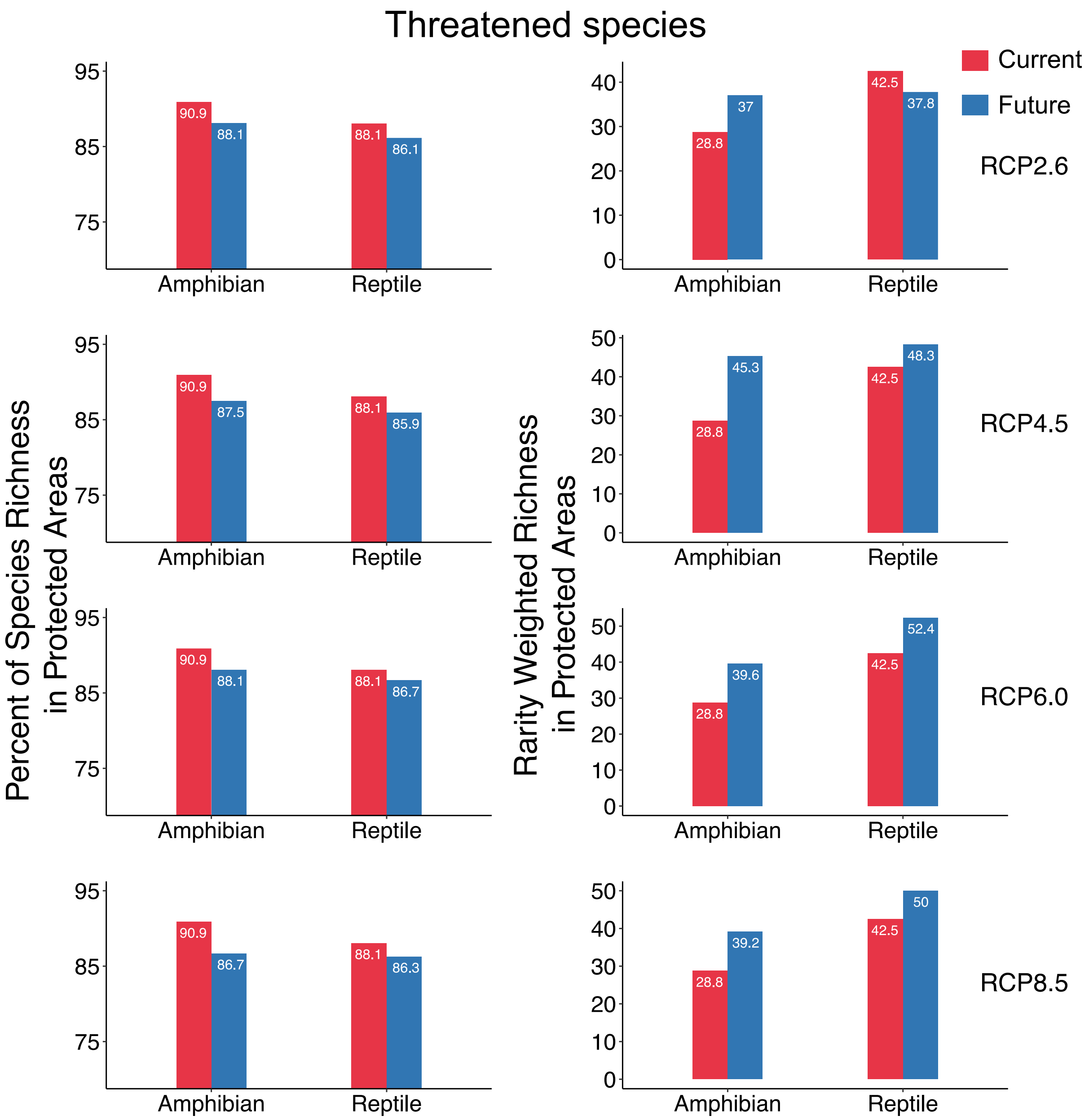

Supplementary Figure 4 Percent of IUCN threatened species and their rarity weighted richness in protected areas (PAs). The first column represents the percent of species in PAs, the second column represents rarity weighted richness in PAs. The first to fourth rows represent RCP 2.6, 4.5, 6.0 and 8.5. The red color indicates current, blue indicates by 2070. We assume future land use remains unchanged for this study.

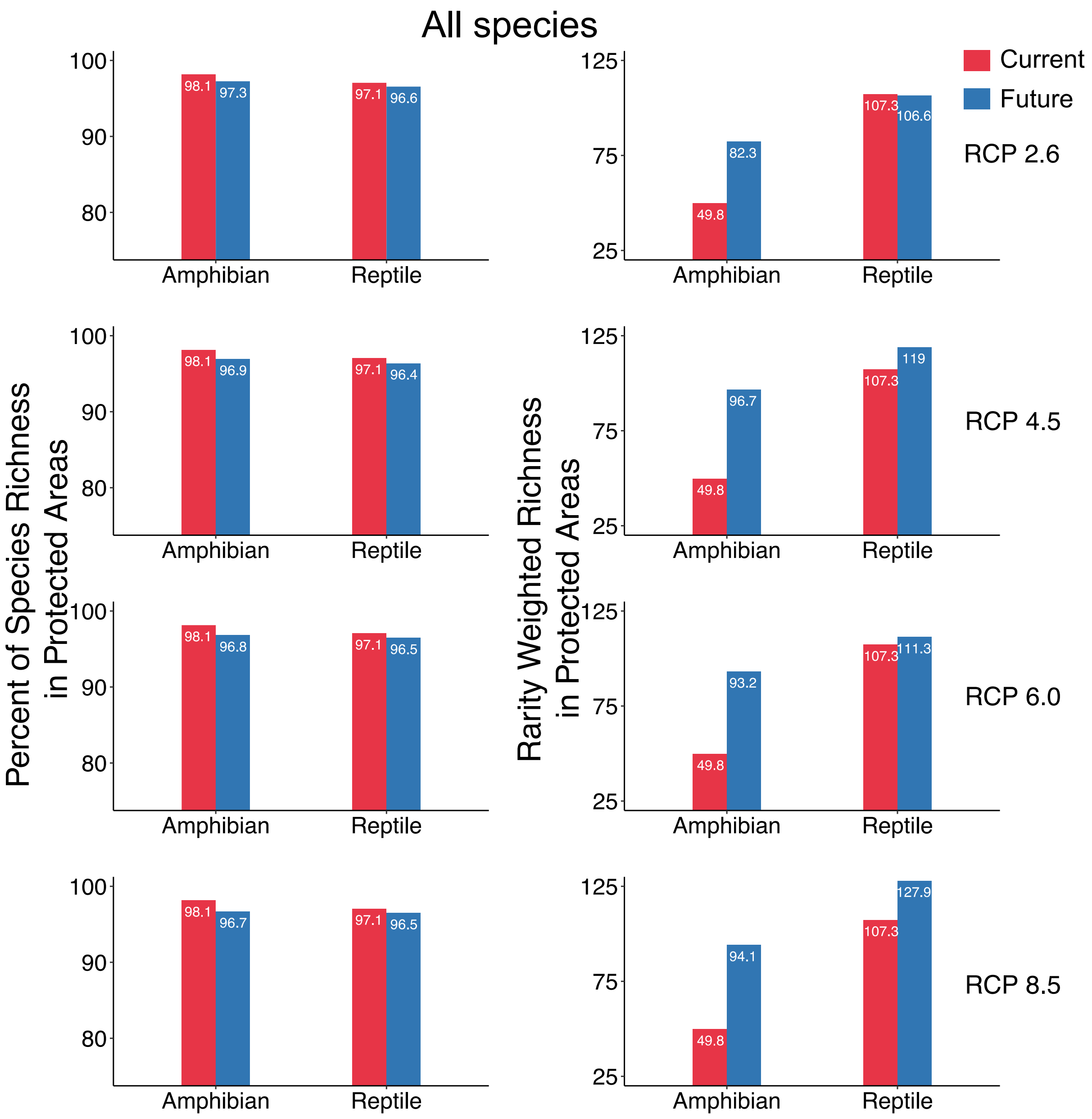

Supplementary Figure 5 Percent of all species and their rarity weighted richness in all protected areas (PAs, Class I to VI). The first column represents the percent of species in PAs, the second column represents rarity weighted richness in PAs. The first to fourth rows represent RCP 2.6, 4.5, 6.0 and 8.5. The red color indicates current, blue indicates by 2070. We assume future land use remains unchanged for this study.

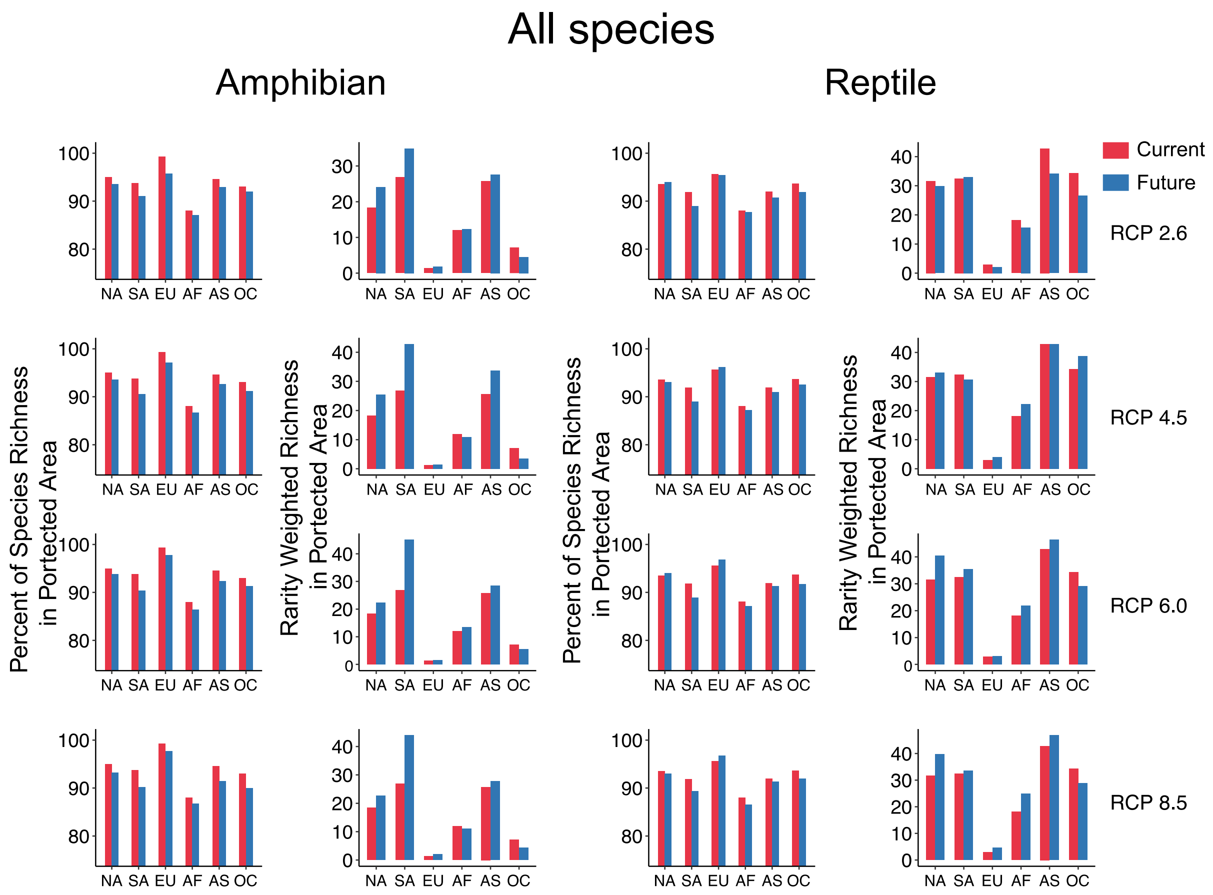

Supplementary Figure 6 Percent of species and rarity weighted richness in protected areas (PAs) in six continents for all species. The first and third columns represent percent of species richness in PAs for amphibians and reptiles; The second and fourth columns represent rarity weighted richness in PAs. The first to fourth rows represent RCP 2.6, 4.5, 6.0 and 8.5. The red color indicates current, blue indicates by 2070. Abbreviations for continents: NA = North America, SA = South America, EU = Europe, AF = Africa, AS = Asia, OC = Oceania. We assume future land use remains unchanged for this study.

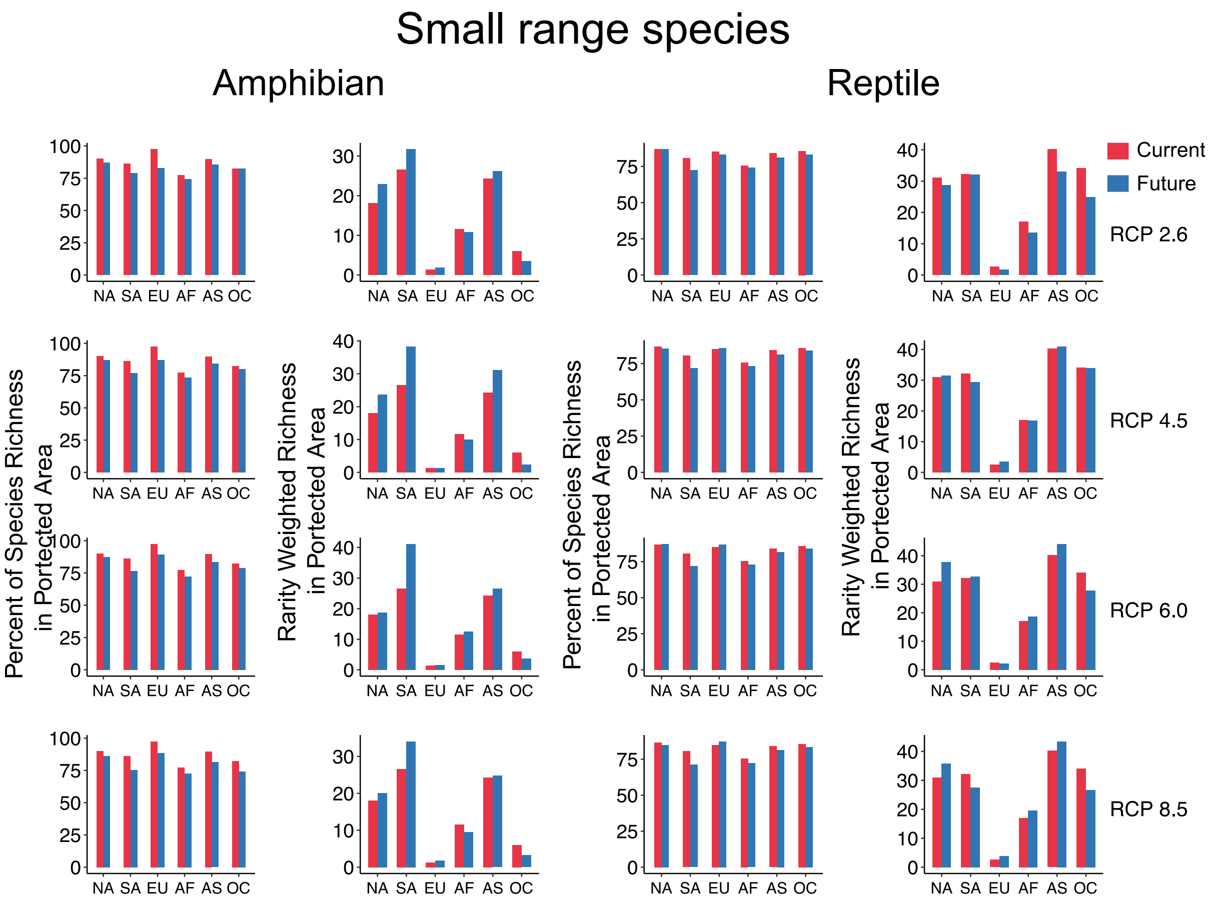

Supplementary Figure 7 Percent of species and rarity weighted richness in protected areas (PAs) in six continents for small range species. The first and third columns represent the percent of species richness in PAs for amphibians and reptiles; The second and fourth columns represent rarity weighted richness in PAs. The first to fourth rows represent RCP 2.6, 4.5, 6.0 and 8.5. The red color indicates current, blue indicates by 2070. Abbreviations for continents: NA = North America, SA = South America, EU = Europe, AF = Africa, AS = Asia, OC = Oceania. We assume future land use remains unchanged for this study.

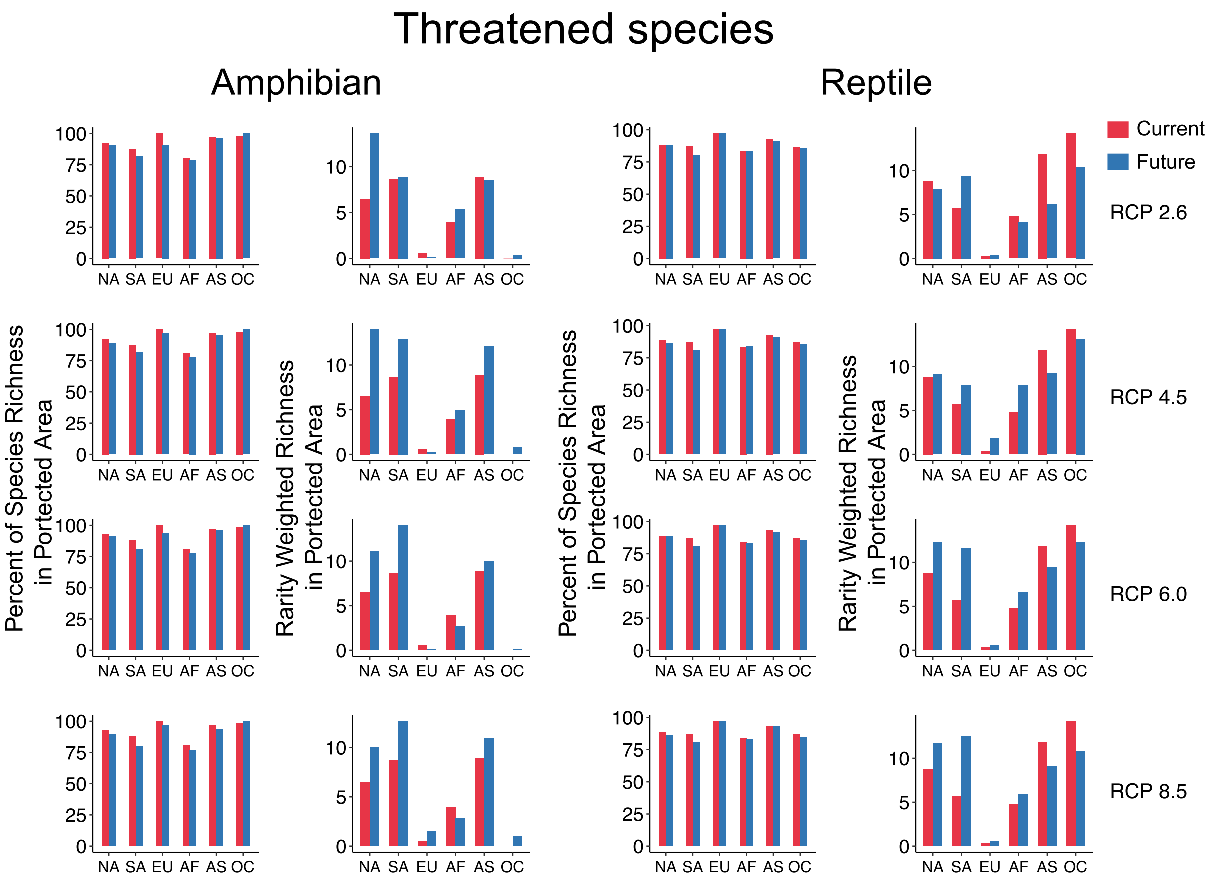

Supplementary Figure 8 Percent of species and rarity weighted richness in protected areas (PAs) in six continents for IUCN threatened species. The first and third columns represent the percent of species richness in PAs for amphibians and reptiles; The second and fourth columns represent rarity weighted richness in PAs. The first to fourth rows represent RCP 2.6, 4.5, 6.0 and 8.5. The red color indicates current, blue indicates by 2070. Abbreviations for continents: NA = North America, SA = South America, EU = Europe, AF = Africa, AS = Asia, OC = Oceania. We assume future land use remains unchanged for this study.

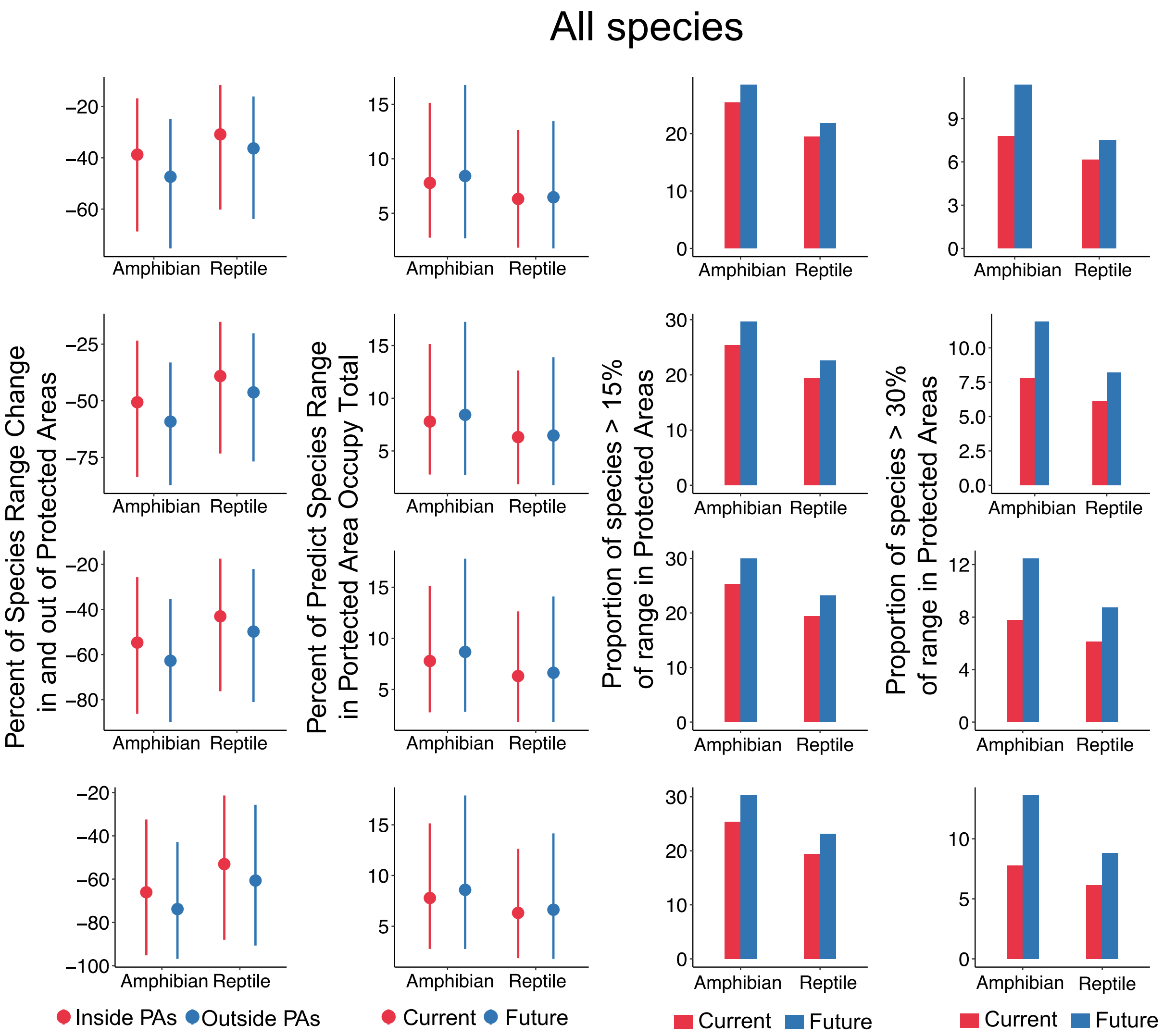

Supplementary Figure 9 Climate change impacts on the percentage of species range (area of habitat) inside and outside protected areas (PAs) by 2070 for all species. The first column represents a percent of species range change inside and outside PAs; the second column represents the percent of predicted species range in PAs at present and by 2070; the third column represents the proportion of species having > 15% of range inside PAs; the fourth column represents the proportion of species having > 30% of range inside PAs. The first to fourth rows represent RCP 2.6, 4.5, 6.0 and 8.5. In first column n = 5399 and 8932 for amphibian and reptiles. In second column n = 5399 and 8932 for amphibian and reptile under current; n = 5040, 4876, 4835 and 4628 for amphibian, 8387, 8190, 8134 and 7834 for reptile under RCP 2.6, 4.5, 6.0 and 8.5, respectively. The points and error bars in the first and second columns represent the medians, the 25% and 75% quantiles. We assume future land use remains unchanged for this study.

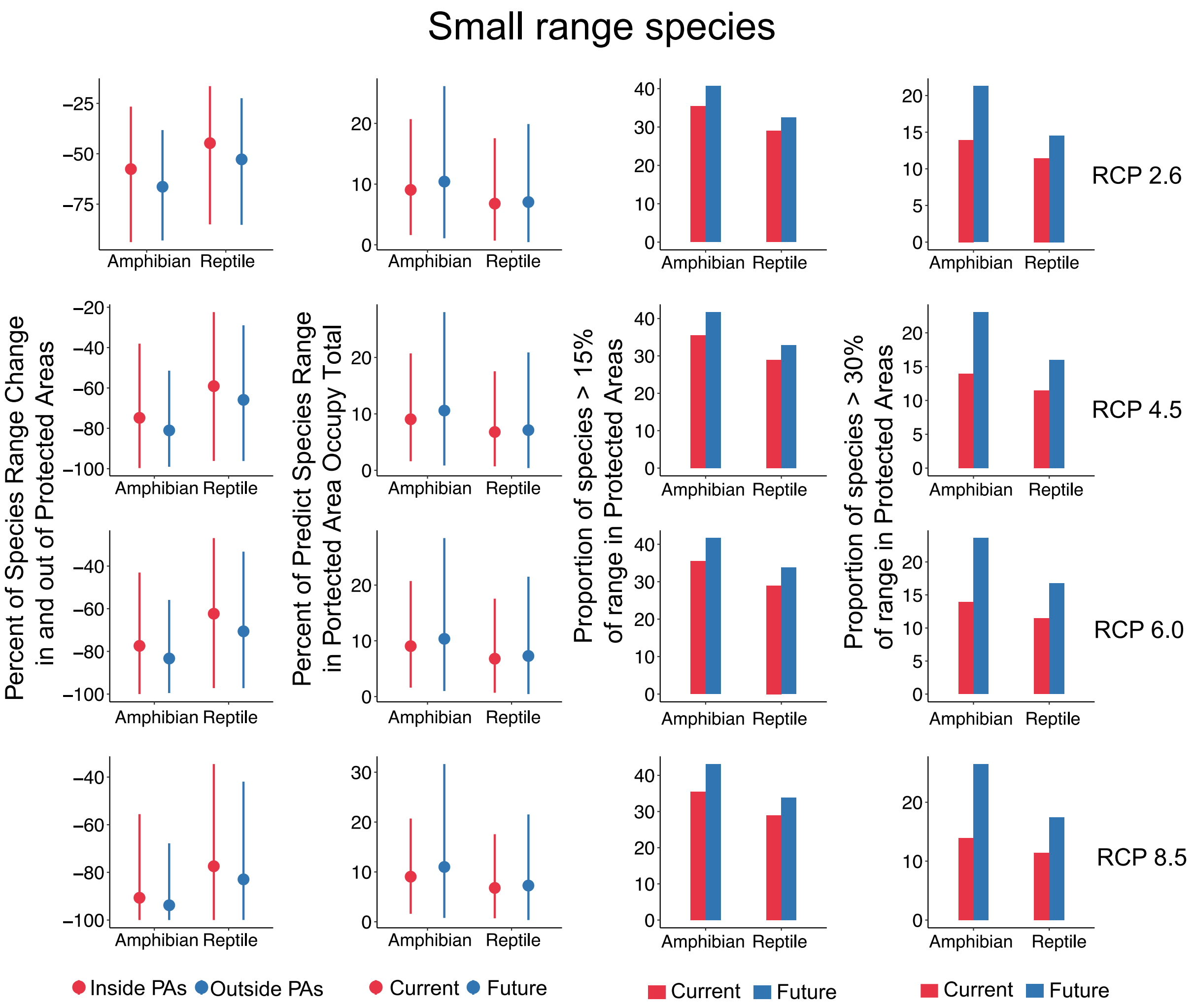

Supplementary Figure 10 Climate change impacts on the percentage of species range (area of habitat) inside and outside protected areas (PAs) by 2070 for small range species. The first column represents the percent of species range change inside and outside PAs; the second column represents the percent of predicted species range in PAs at present and by 2070; the third column represents the proportion of species having > 15% of range inside PAs; the fourth column represents the proportion of species having > 30% of range inside PAs. The first to fourth rows represent RCP 2.6, 4.5, 6.0 and 8.5. In first column n = 2697 and 4435 for amphibian and reptiles. In second column n = 2697 and 4435 for amphibian and reptile under current; n = 2354, 2194, 2161 and 1967 for amphibian, 3897, 3707, 3660 and 3383 for reptile under RCP 2.6, 4.5, 6.0 and 8.5, respectively. The points and error bars in the first and second columns represent the medians, the 25% and 75% quantiles. We assume future land use remains unchanged for this study.

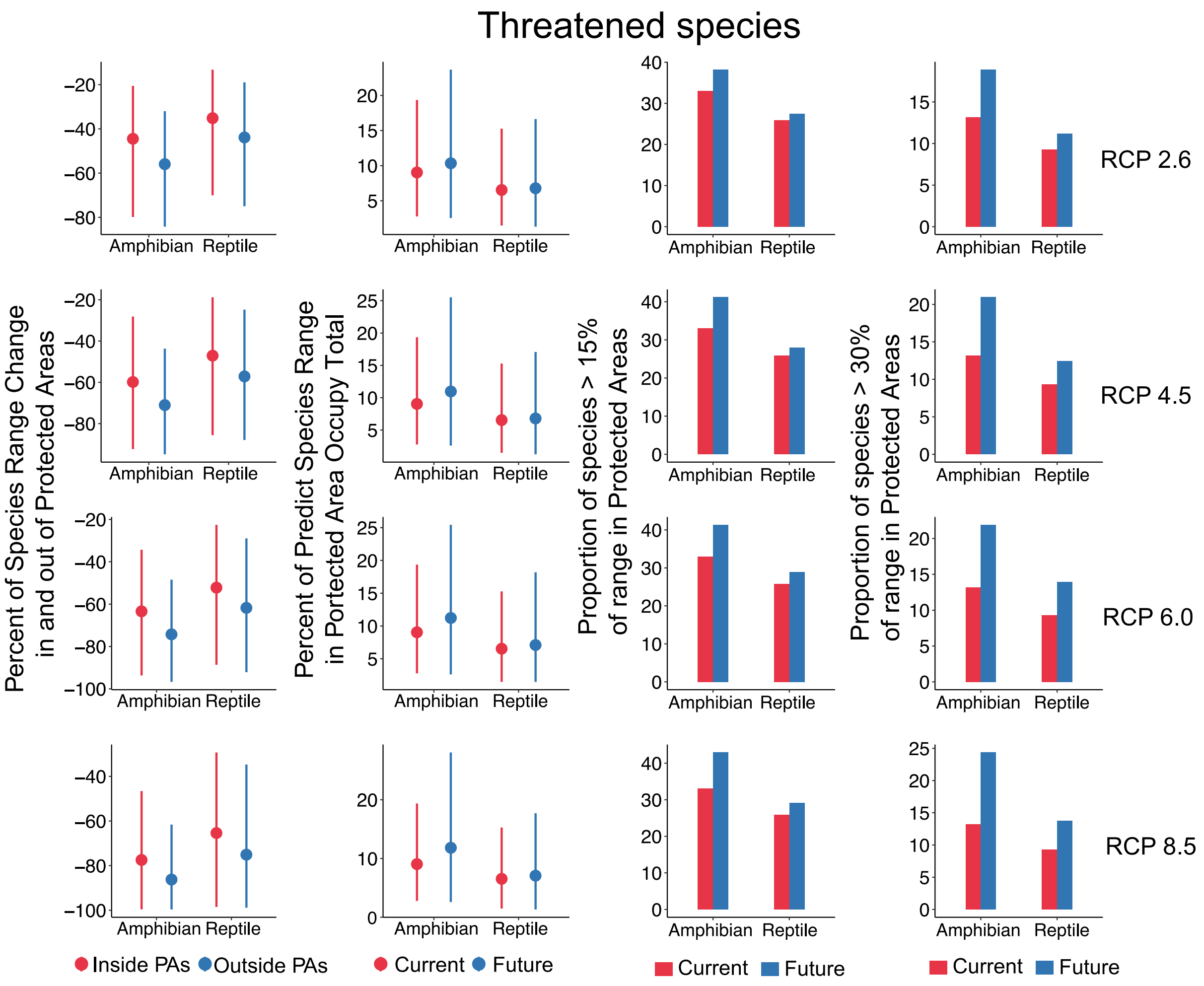

Supplementary Figure 11 Climate change impacts on the percentage of species range (area of habitat) inside and outside protected areas (PAs) by 2070 for IUCN threatened species. The first column represents the percent of species range change inside and outside PAs; the second column represents the percent of predicted species range in PAs at present and by 2070; the third column represents the proportion of species having > 15% of range inside PAs; the fourth column represents the proportion of species having > 30% of range inside PAs. The first to fourth rows represent RCP 2.6, 4.5, 6.0 and 8.5. In first column *n* = 1275 and 1474 for amphibian and reptiles. In second column *n* = 1275 and 1474 for amphibian and reptile under current; *n* = 1160, 1112, 1091 and 1022 for amphibian, 1335, 1288, 1260 and 1181 for reptile under RCP 2.6, 4.5, 6.0 and 8.5, respectively. The points and error bars in the first and second columns represent the medians, the 25% and 75% quantiles. We assume future land use remains unchanged for this study.

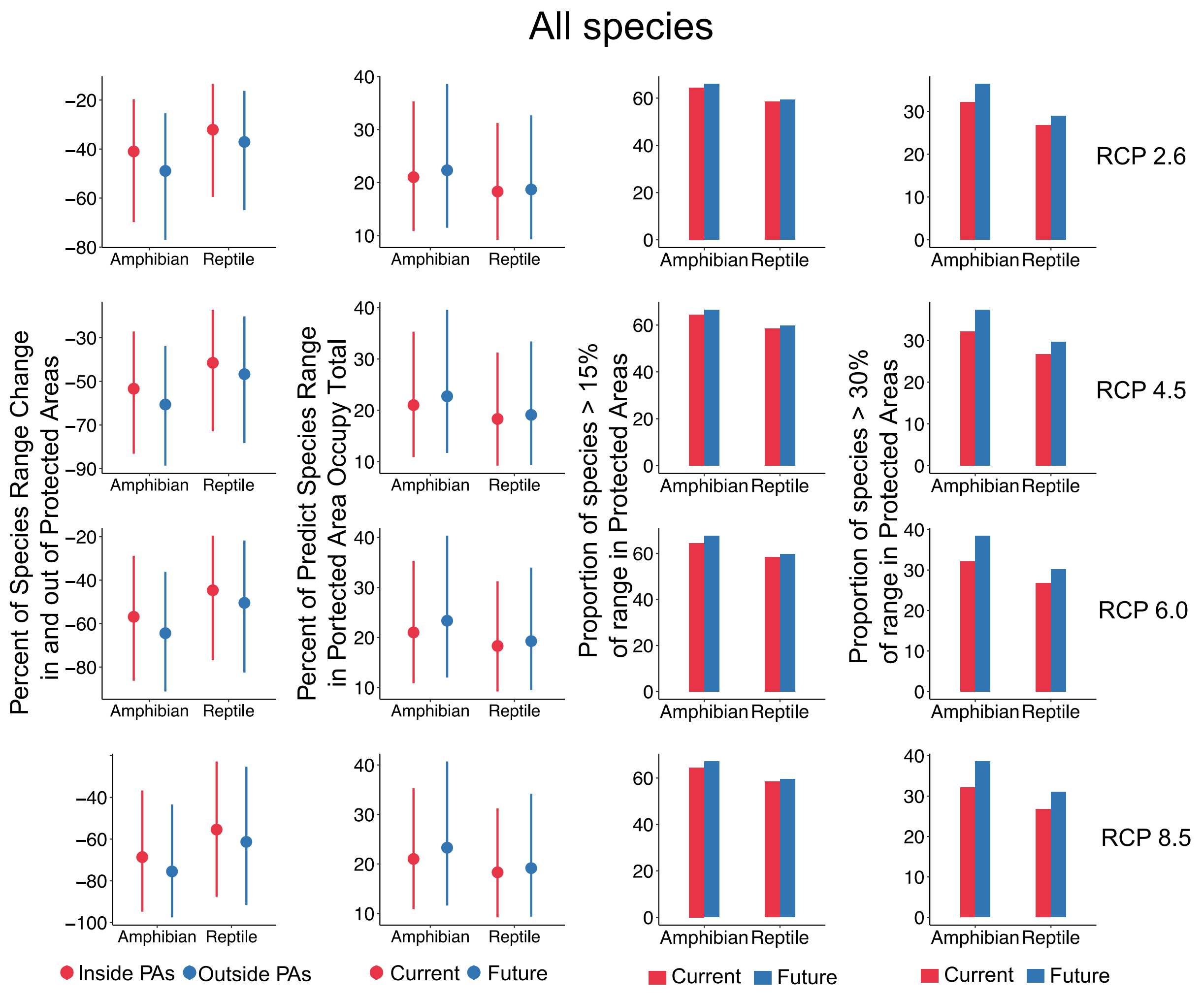

Supplementary Figure 12 Climate change impacts on the percentage of species range (area of habitat) inside and outside all protected areas (PAs, Class I to VI) by 2070 for all species (Class I to VI). The first column represents a percent of species range change inside and outside PAs; the second column represents the percent of predicted species range in PAs at present and by 2070; the third column represents the proportion of species having > 15% of range inside PAs; the fourth column represents the proportion of species having > 30% of range inside PAs. The first to fourth rows represent RCP 2.6, 4.5, 6.0 and 8.5. In first column n = 5399 and 8932 for amphibian and reptiles. In second column n = 5399 and 8932 for amphibian and reptile under current; n = 5040, 4876, 4835 and 4628 for amphibian, 8387, 8190, 8134 and 7834 for reptile under RCP 2.6, 4.5, 6.0 and 8.5, respectively. The points and error bars in the first and second columns represent the medians, the 25% and 75% quantiles. We assume future land use remains unchanged for this study.

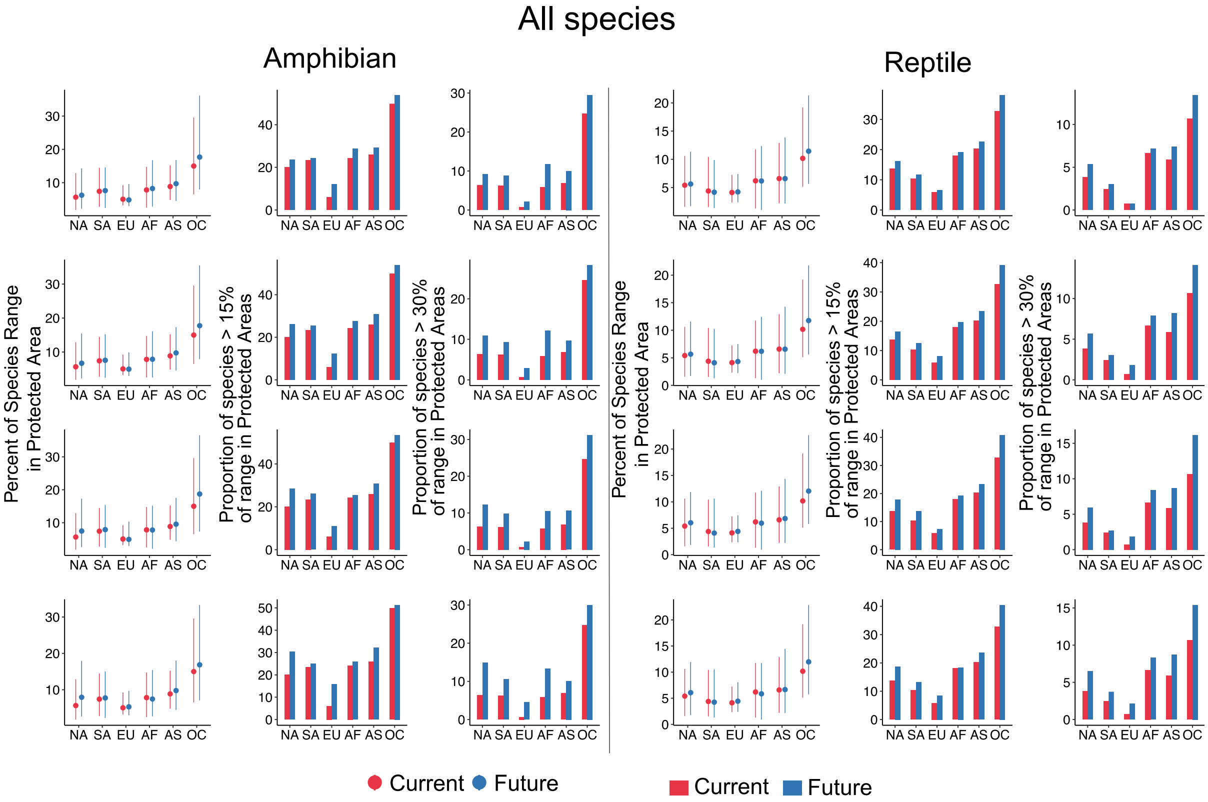

Supplementary Figure 13 Climate change impacts on the percentage of species range (area of habitat) in protected areas (PAs) in six continents for all species. The first and four columns represent the percentage of species range in PAs for amphibians and reptiles; the second and fifth column represents the proportion of species having > 15% of range inside PAs for amphibians and reptiles; the third and sixth column represents the proportion of species having > 30% of range inside PAs. The first to fourth rows represent RCP 2.6, 4.5, 6.0 and 8.5. The red color indicates current, blue indicates by 2070. Abbreviations for continents: NA = North America, SA = South America, EU = Europe, AF = Africa, AS = Asia, OC = Oceania. In first column *n* = 1275, 1906, 148, 809, 1272, 357 for six continents under current; 1201, 1780, 142, 754, 1190, 337 for six continents under RCP 2.6; 1168, 1715, 138, 729, 1163, 329 under RCP 4.5; 1143, 1714, 135, 724, 1160, 324 under RCP 6.0; 1105, 1642, 133, 690, 1118, 310 under RCP 8.5. In fourth column *n* = 2149, 1763, 410, 1818, 2535, 1521 for six continents under current; 2042, 1674, 397, 1721, 2398, 1389 for six continents under RCP 2.6; 2020, 1620, 387, 1698, 2334, 1356 under RCP 4.5; 1995, 1641, 380, 1671, 2330, 1331 under RCP 6.0; 1955, 1552, 377, 1635, 2253, 1273 under RCP 8.5. The points and error bars in the first and fourth columns represent the medians, the 25% and 75% quantiles. We assume future land use remains unchanged for this study.

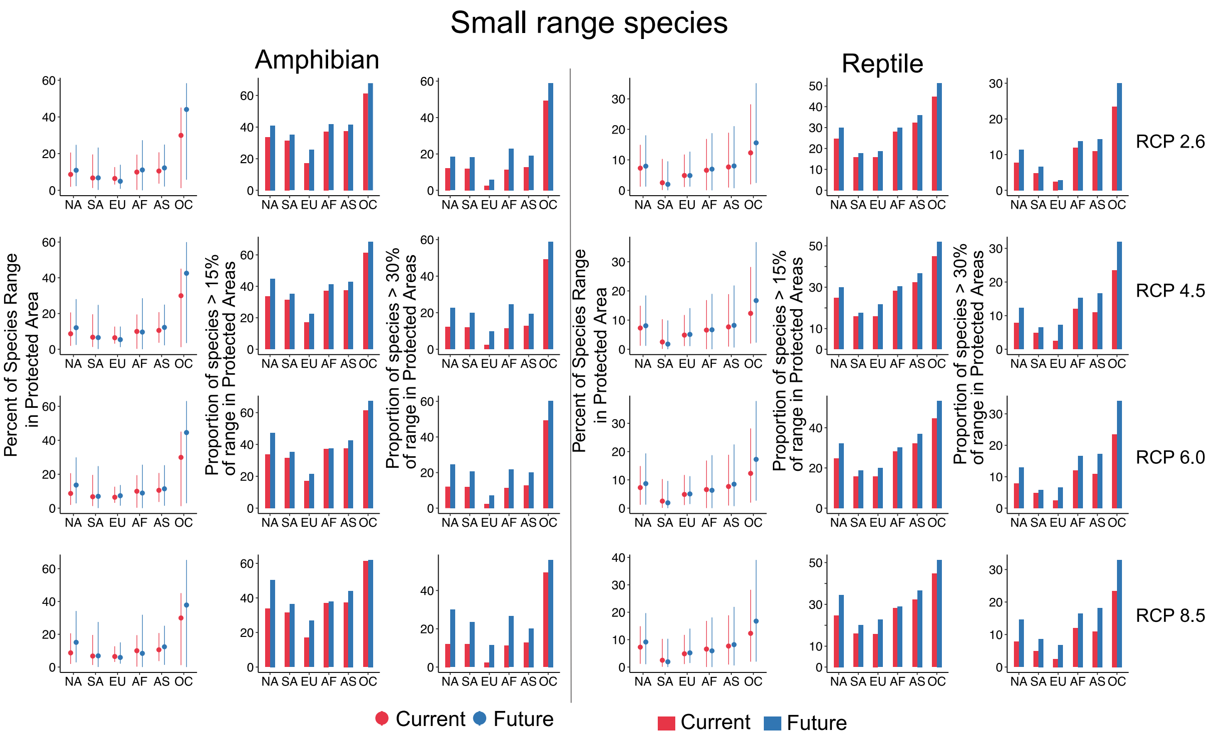

Supplementary Figure 14 Climate change impacts on the percentage of species range (area of habitat) in protected areas (PAs) in six continents for small range species. The first and four columns represent the percentage of species range in PAs for amphibians and reptiles; the second and fifth column represents the proportion of species having > 15% of range inside PAs for amphibians and reptiles; the third and sixth column represents the proportion of species having > 30% of range inside PAs. The first to fourth rows represent RCP 2.6, 4.5, 6.0 and 8.5. The red color indicates current, blue indicates by 2070. Abbreviations for continents: NA = North America, SA = South America, EU = Europe, AF = Africa, AS = Asia, OC = Oceania. In first column n = 652, 855, 41, 415, 624, 142 for six continents under current; 581, 732, 35, 362, 548, 124 for six continents under RCP 2.6; 550, 669, 31, 337, 521, 116 under RCP 4.5; 533, 666, 28, 332, 518, 113 under RCP 6.0; 500, 597, 26, 301, 476, 100 under RCP 8.5. In fourth column n = 1048, 742, 120, 853, 1265, 661 for six continents under current; 941, 656, 107, 756, 1128, 533 for six continents under RCP 2.6; 920, 606, 97, 733, 1064, 502 under RCP 4.5; 900, 624, 90, 706, 1061, 484 under RCP 6.0; 862, 542, 88, 672, 985, 437 under RCP 8.5. The points and error bars in the first and fourth columns represent the medians, the 25% and 75% quantiles. We assume future land use remains unchanged for this study.

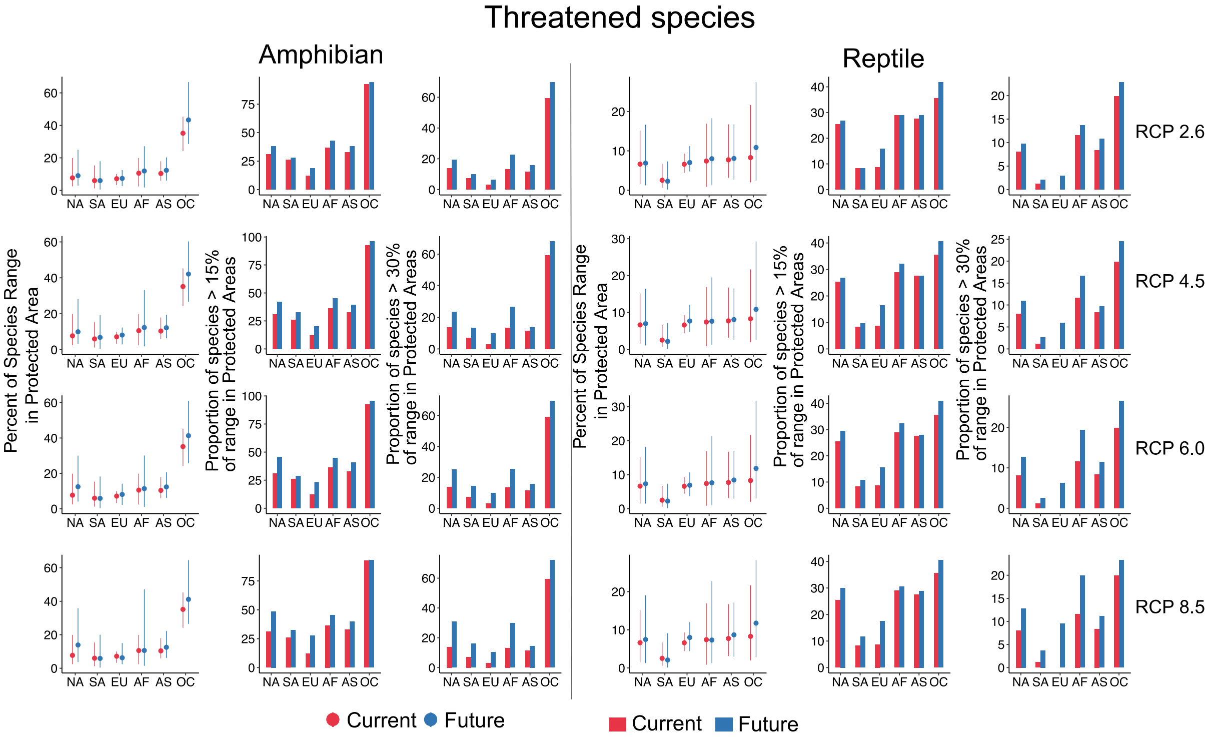

1. **Supplementary Figure 15 Climate change impacts on the percentage of species range (area of habitat) in protected areas (PAs) in six continents for IUCN threatened species.** The first and four columns represent the percentage of species range in PAs for amphibians and reptiles; the second and fifth column represents the proportion of species having > 15% of range inside PAs for amphibians and reptiles; the third and sixth column represents the proportion of species having > 30% of range inside PAs. The first to fourth rows represent RCP 2.6, 4.5, 6.0 and 8.5. The red color indicates current, blue indicates by 2070. Abbreviations for continents: NA = North America, SA = South America, EU = Europe, AF = Africa, AS = Asia, OC = Oceania. In first column *n* = 400, 334, 33, 181, 290, 54 for six continents under current; 364, 299, 32, 163, 267, 53 for six continents under RCP 2.6; 346, 290, 30, 153, 262, 50 under RCP 4.5; 334, 289, 30, 149, 260, 46 under RCP 6.0; 312, 269, 29, 141, 249, 43 under RCP 8.5. In fourth column *n* = 397, 254, 69, 293, 395, 191 for six continents under current; 361, 239, 69, 263, 371, 153 for six continents under RCP 2.6; 354, 227, 67, 258, 359, 143 under RCP 4.5; 345, 232, 64, 247, 350, 139 under RCP 6.0; 336, 214, 63, 236, 332, 116 under RCP 8.5. The points and error bars in the first and fourth columns represent the medians, the 25% and 75% quantiles. We assume future land use remains unchanged for this study.

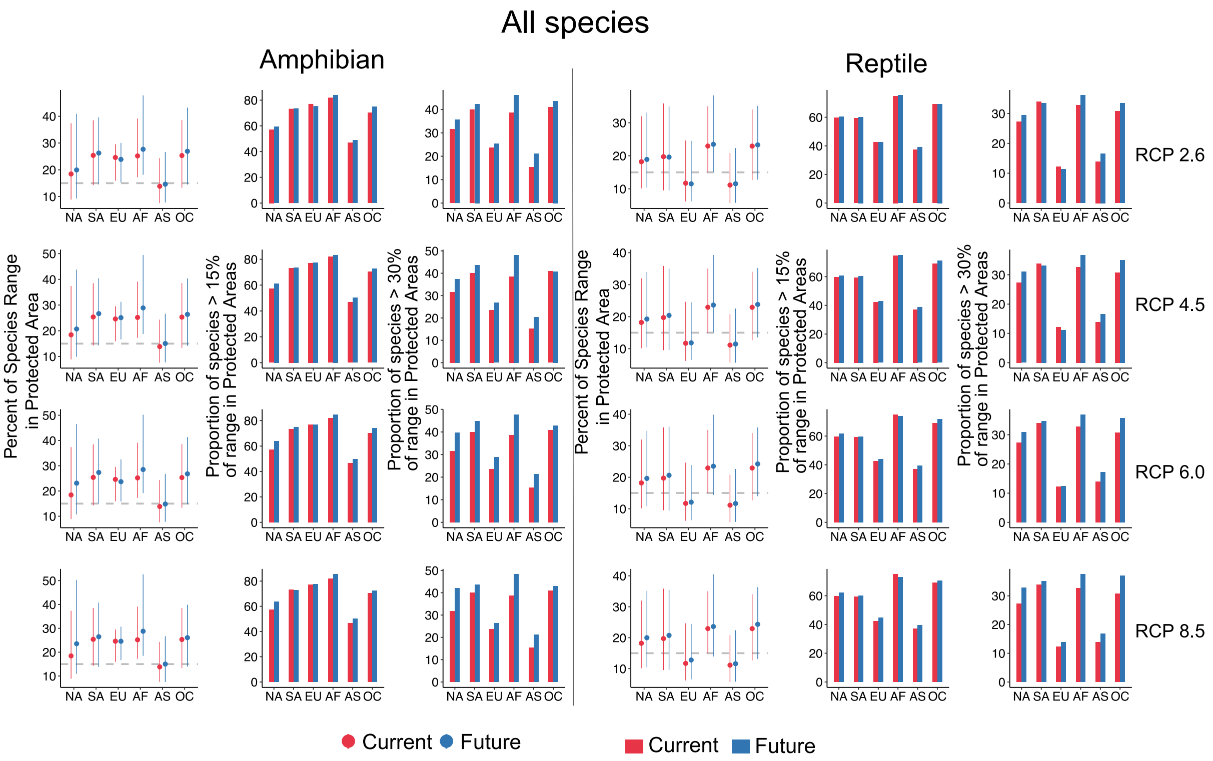

Supplementary Figure 16 Climate change impacts on the percentage of species range (area of habitat) in all protected areas (PAs, Class I to VI) in six continents for all species. The first and four columns represent the percentage of species range in PAs for amphibians and reptiles; the second and fifth column represents the proportion of species having > 15% of range inside PAs for amphibians and reptiles; the third and sixth column represents the proportion of species having > 30% of range inside PAs. The first to fourth rows represent RCP 2.6, 4.5, 6.0 and 8.5. The red color indicates current, blue indicates by 2070. Abbreviations for continents: NA = North America, SA = South America, EU = Europe, AF = Africa, AS = Asia, OC = Oceania. In first column *n* = 1275, 1906, 148, 809, 1272, 357 for six continents under current; 1201, 1780, 142, 754, 1190, 337 for six continents under RCP 2.6; 1168, 1715, 138, 729, 1163, 329 under RCP 4.5; 1143, 1714, 135, 724, 1160, 324 under RCP 6.0; 1105, 1642, 133, 690, 1118, 310 under RCP 8.5. In fourth column *n* = 2149, 1763, 410, 1818, 2535, 1521 for six continents under current; 2042, 1674, 397, 1721, 2398, 1389 for six continents under RCP 2.6; 2020, 1620, 387, 1698, 2334, 1356 under RCP 4.5; 1995, 1641, 380, 1671, 2330, 1331 under RCP 6.0; 1955, 1552, 377, 1635, 2253, 1273 under RCP 8.5. The points and error bars in the first and fourth columns represent the medians, the 25% and 75% quantiles. We assume future land use remains unchanged for this study.

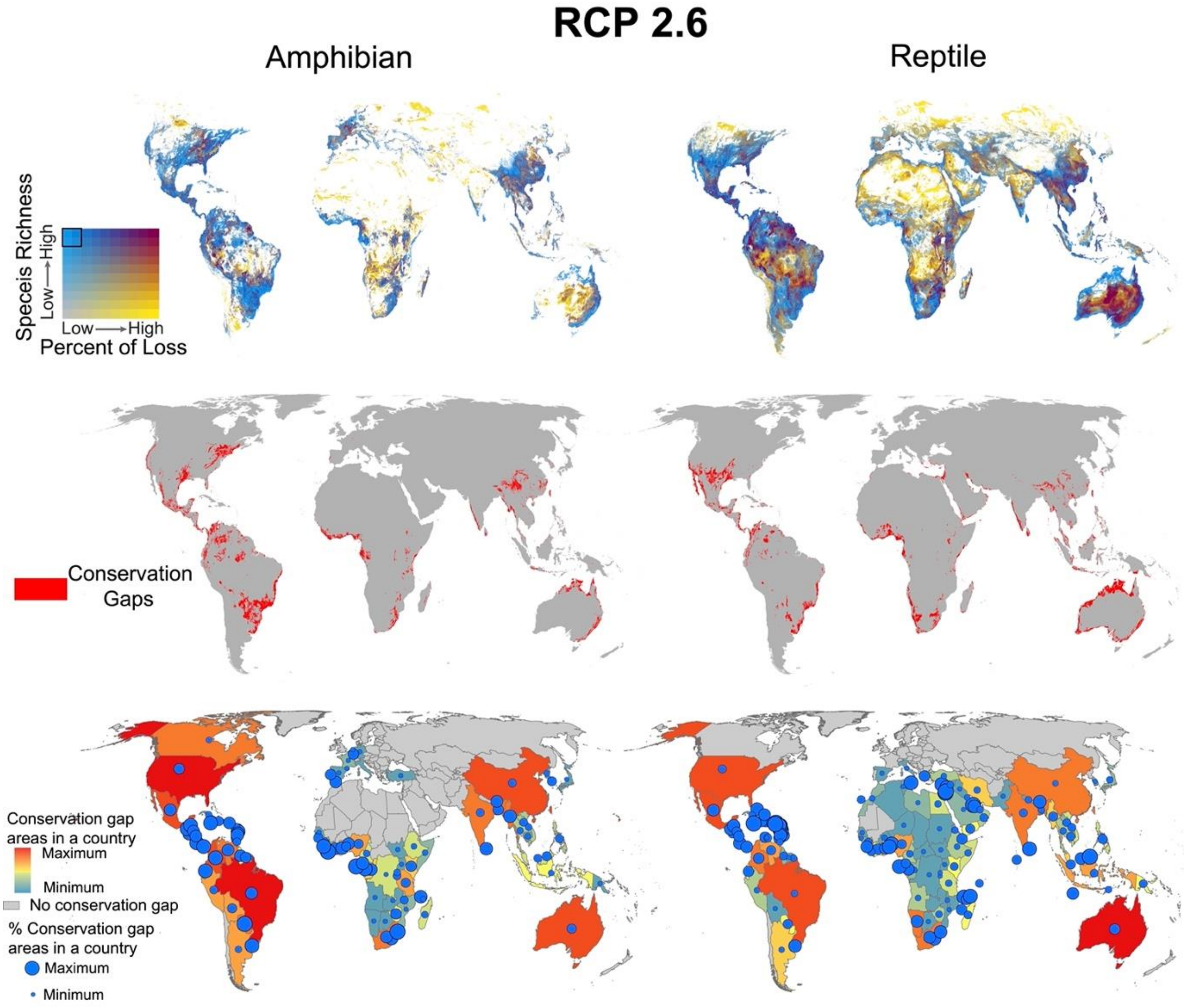

Supplementary Figure 17 Conservation priority and conservation gaps for global herpetofauna by 2070 (RCP 2.6). The first row represents the bivariate maps showing species richness versus percent of species loss for amphibians and reptiles. Each color change means a 10% quantile shift in either variable. Noteworthy, areas in blue represent areas that currently fall into the top species richness category and that are expected to suffer a low percent of species loss due to climate change by 2070; in short, they are climate-robust areas of high species richness. The second row represents conservation gaps (areas falling into the top 20% in terms of species richness and the bottom 20% in terms of future species loss due to climate change, yet fall outside the PA network) of amphibians and reptiles. The third row represents conservation gaps for countries. Colors represent area of conservation gaps in countries, circle sizes represent the percentage of conservation gap area with respect to the land area in countries. We assume future land use remains unchanged for this study.

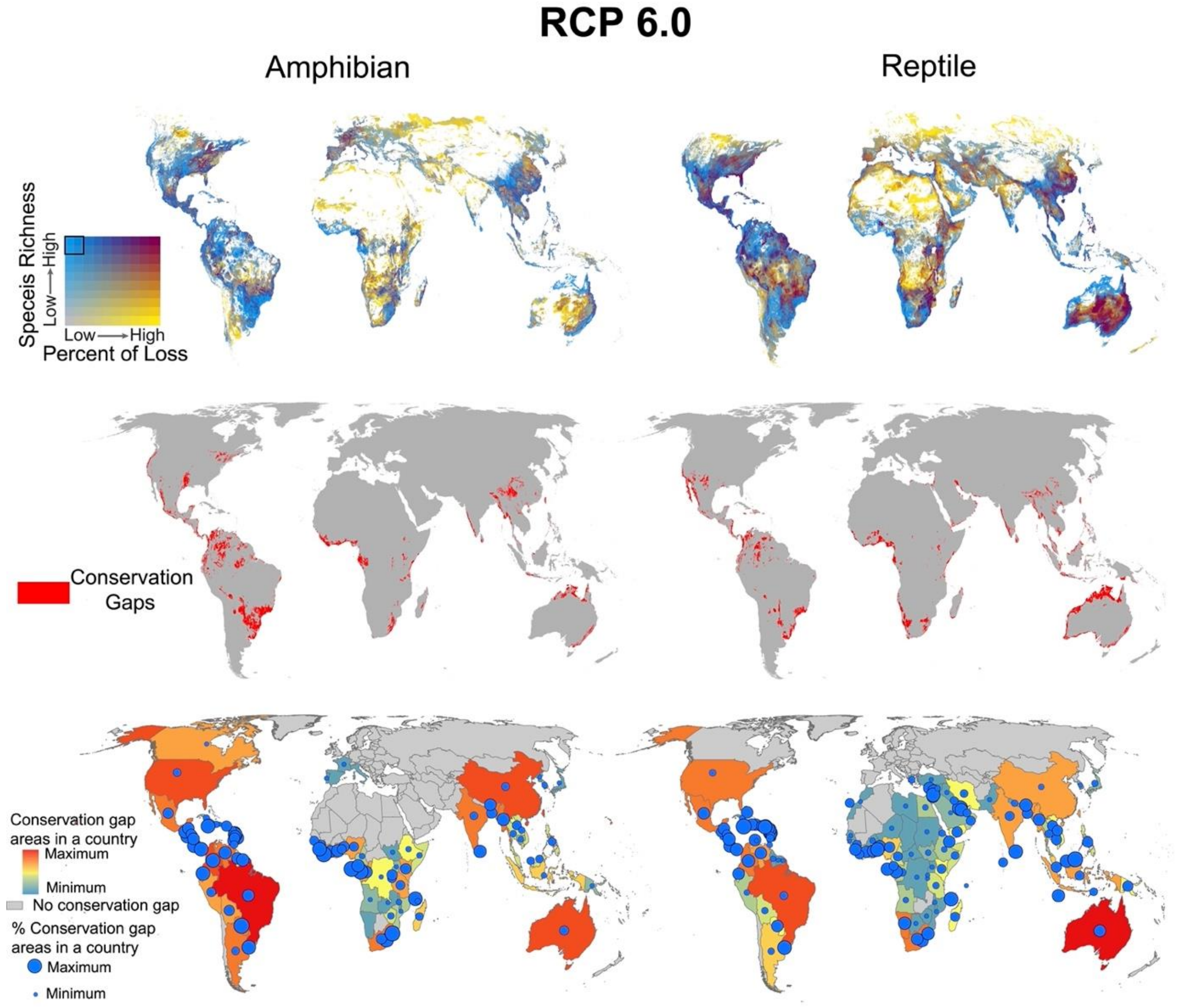

Supplementary Figure 18 Conservation priority and conservation gaps for global herpetofauna by 2070 (RCP 6.0). The first row represents the bivariate maps showing species richness versus percent of species loss for amphibians and reptiles. Each color change means a 10% quantile shift in either variable. Noteworthy, areas in blue represent areas that currently fall into the top species richness category and that are expected to suffer a low percent of species loss due to climate change by 2070; in short, they are climate-robust areas of high species richness. The second row represents conservation gaps (areas falling into the top 20% in terms of species richness and the bottom 20% in terms of future species loss due to climate change, yet fall outside the PA network) of amphibians and reptiles. The third row represents conservation gaps for countries. Colors represent area of conservation gaps in countries, circle sizes represent the percentage of conservation gap area with respect to the land area in countries. We assume future land use remains unchanged for this study.

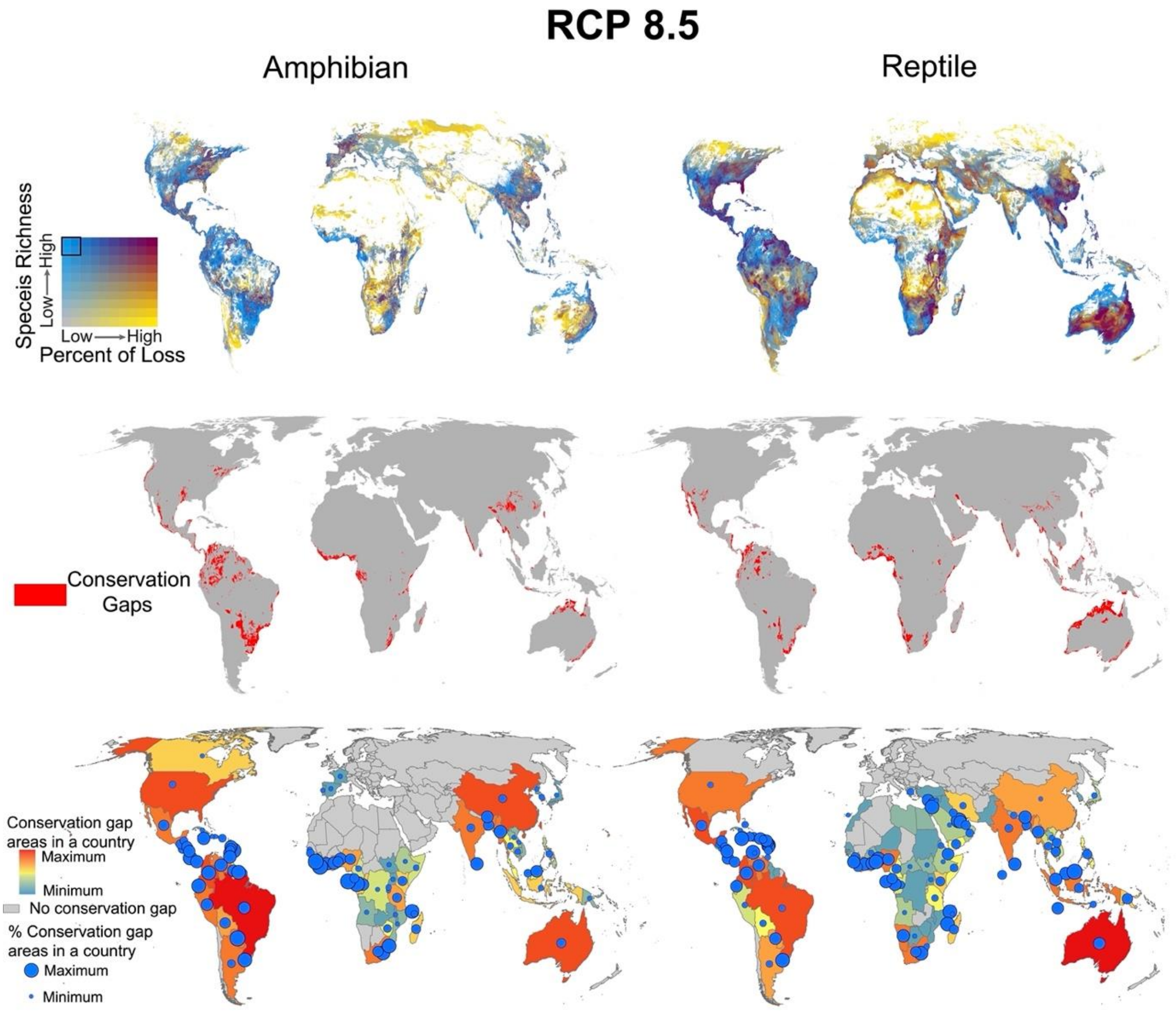

Supplementary Figure 19 Conservation priority and conservation gaps for global herpetofauna by 2070 (RCP 8.5). The first row represents the bivariate maps showing species richness versus percent of species loss for amphibians and reptiles. Each color change means a 10% quantile shift in either variable. Noteworthy, areas in blue represent areas that currently fall into the top species richness category and that are expected to suffer a low percent of species loss due to climate change by 2070; in short, they are climate-robust areas of high species richness. The second row represents conservation gaps (areas falling into the top 20% in terms of species richness and the bottom 20% in terms of future species loss due to climate change, yet fall outside the PA network) of amphibians and reptiles. The third row represents conservation gaps for countries. Colors represent area of conservation gaps in countries, circle sizes represent the percentage of conservation gap area with respect to the land area in countries. We assume future land use remains unchanged for this study.

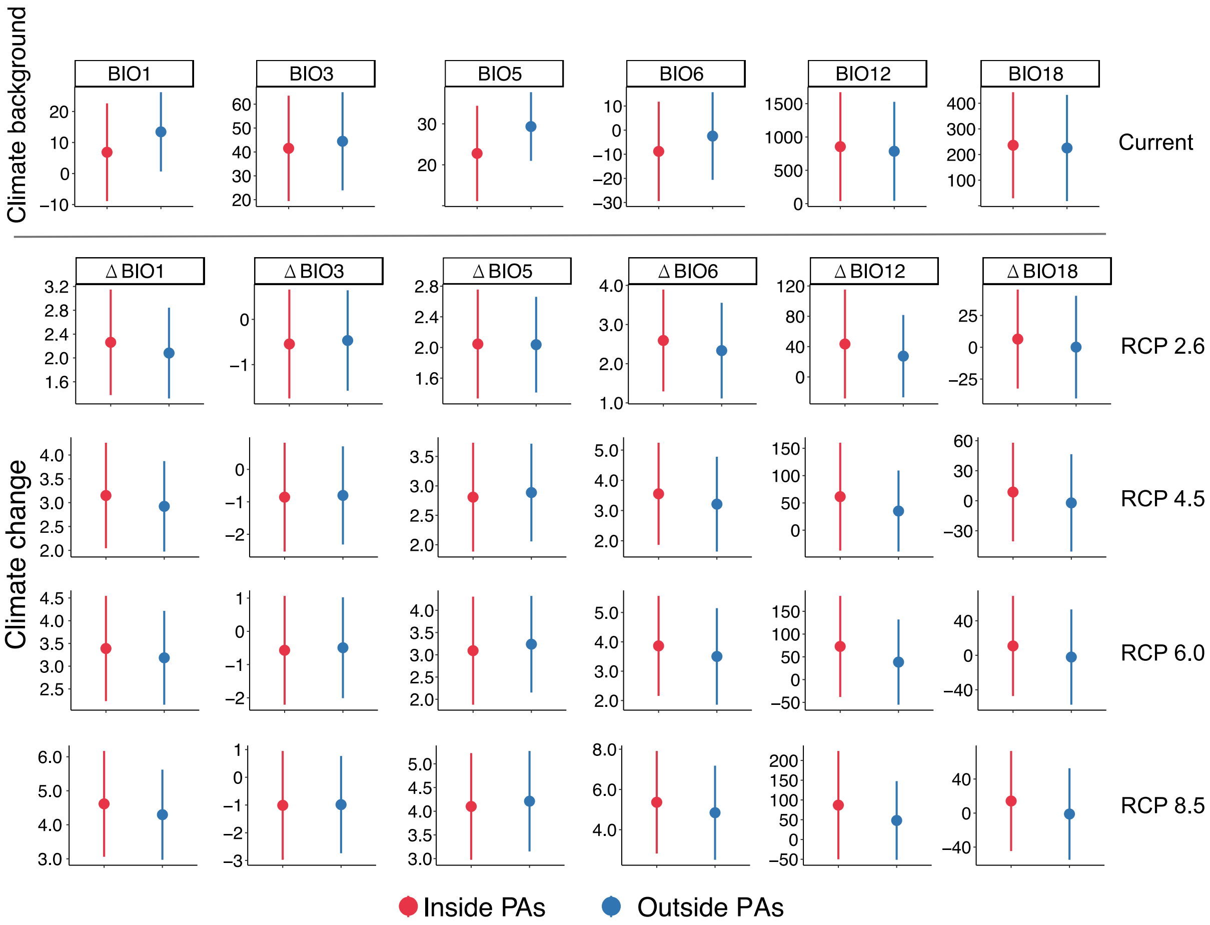

Supplementary Figure 20 Climate and climate change inside and outside protected areas (PAs). *n* = 227234 and 1125139 for inside PAs and outside PAs. The first row represents the climate background, and the second to fifth row represents the change of related climate between past and future under RCP 2.6, 4.5, 6.0 and 8.5 scenarios. BIO1, annual mean temperature; BIO3, Isothermality (Mean Diurnal Range/Temperature Annual Range); BIO5, the max temperature of the warmest month; BIO6, min temperature of the coldest month; BIO12, mean annual precipitation; BIO18, precipitation of warmest quarter. Δ indicates the change of related climate variables. The point and error bar are represented by the mean ±SD.

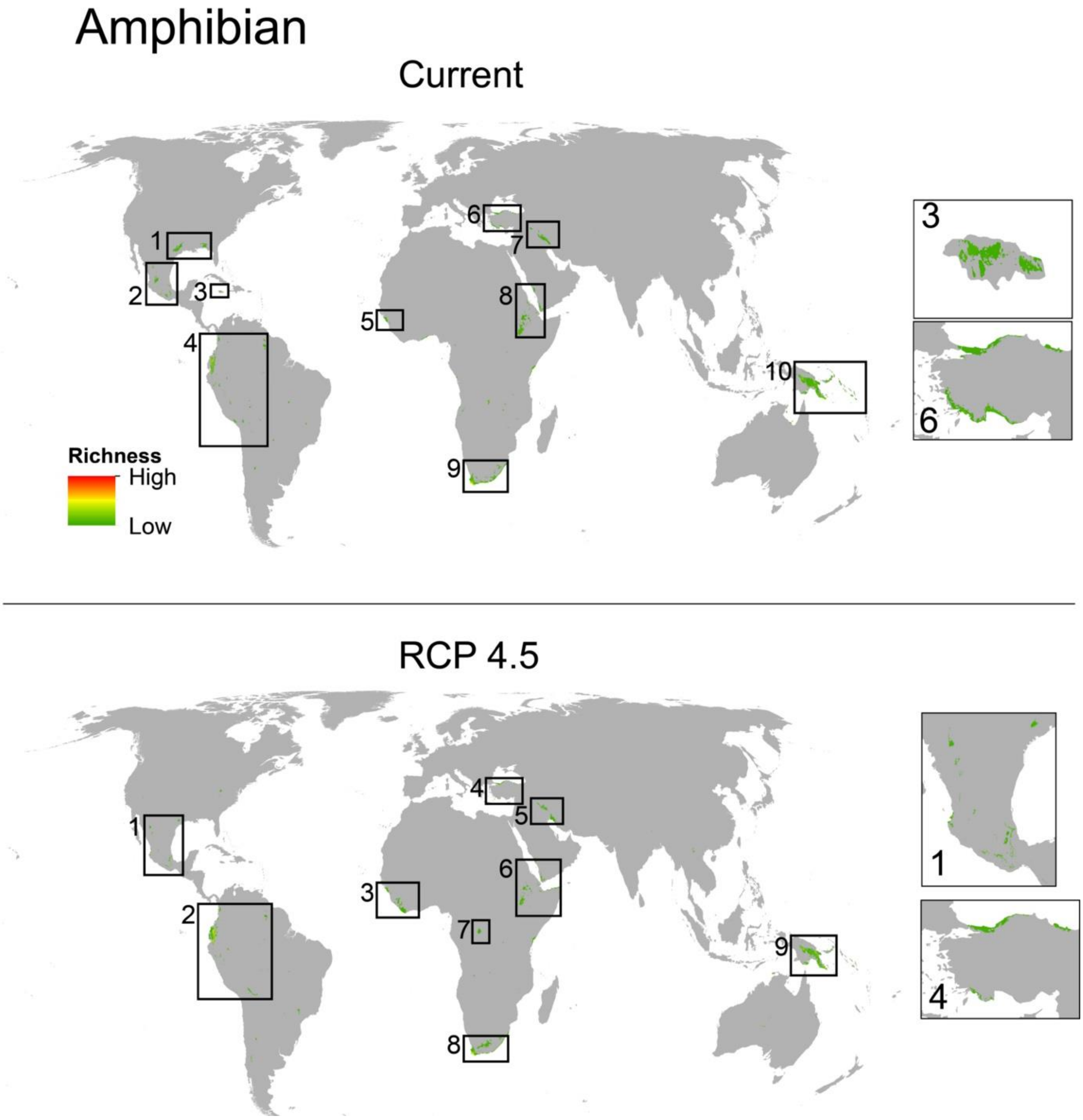

Supplementary Figure 21 The spatial distribution of amphibian species whose whole range are not inside protected areas (PAs).

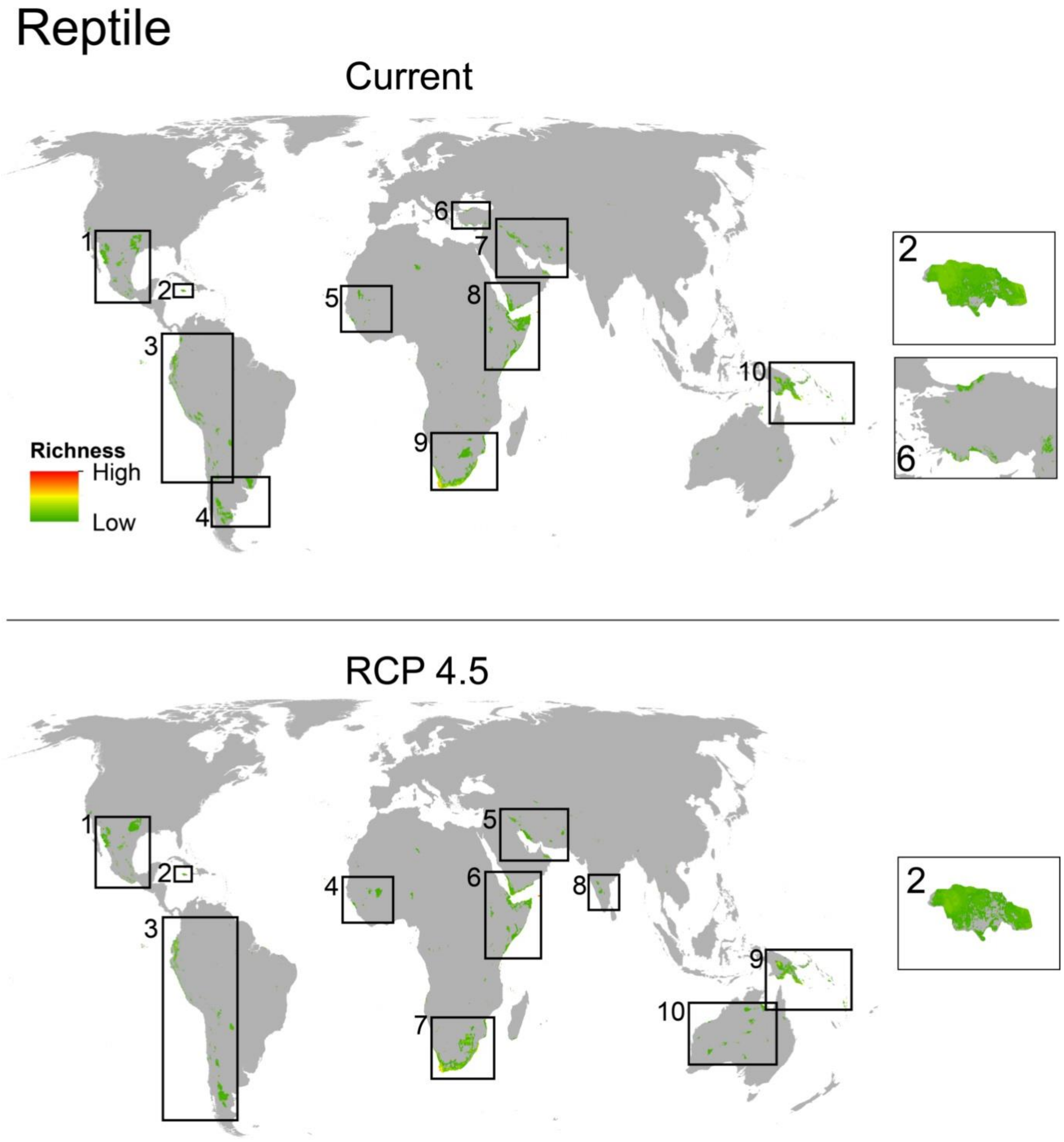

Supplementary Figure 22 The spatial distribution of reptile species whose whole range are not inside protected areas (PAs).

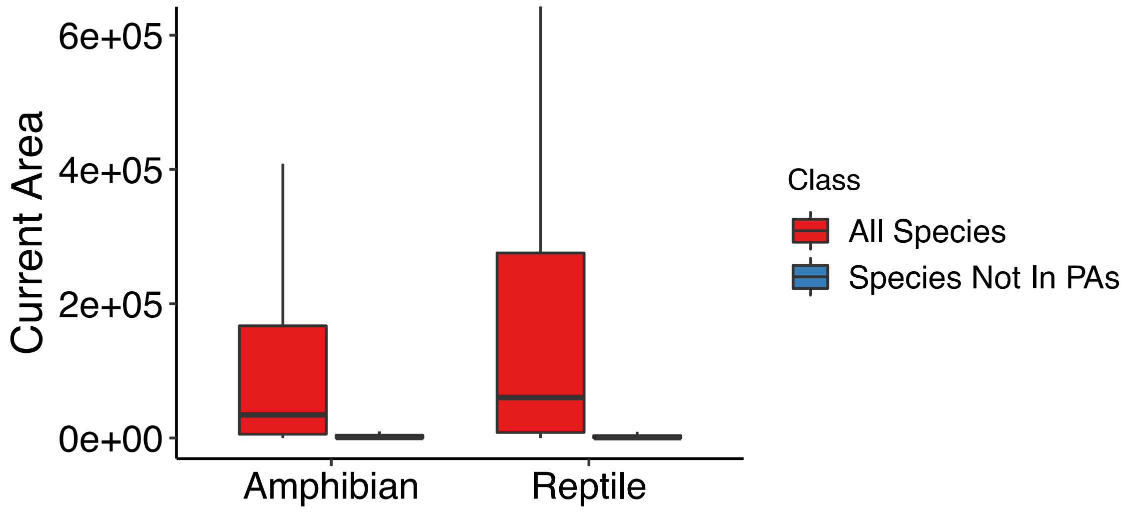

Supplementary Figure 23 Distribution of species range for all species and species not in protected areas (PAs). *n* = 5399 and 365 for all amphibian species and amphibian species not in PAs, 8932 and 770 for all reptile species and reptile species not in PAs. Boxes are bounded within the first and third quartiles, medians represented by thick horizontal lines within each box and, whiskers extending to the minimum and maximum values that do not exceed 1.5 times the interquartile range from the median.

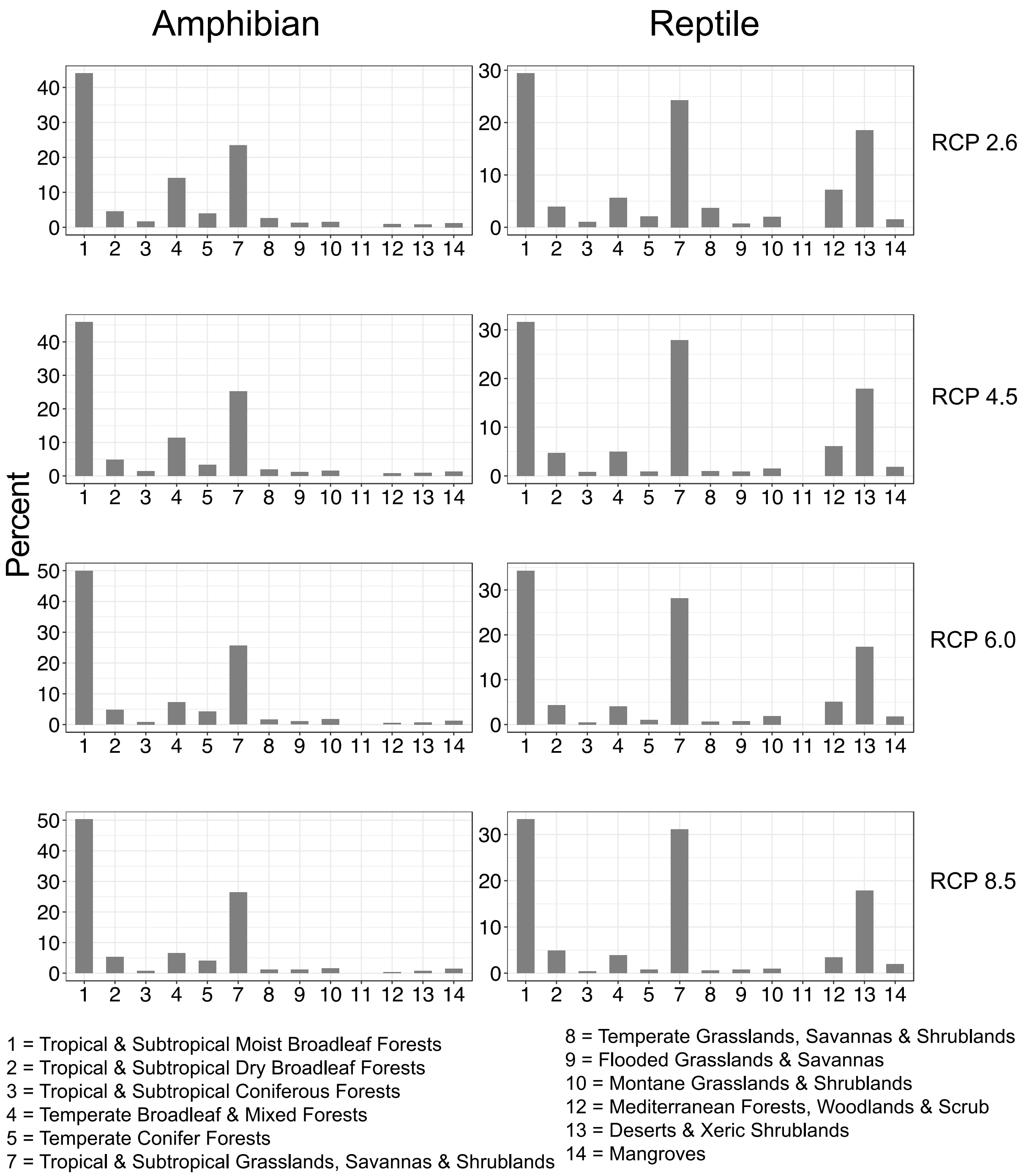

Supplementary Figure 24 Percent of biome types in conservation gaps

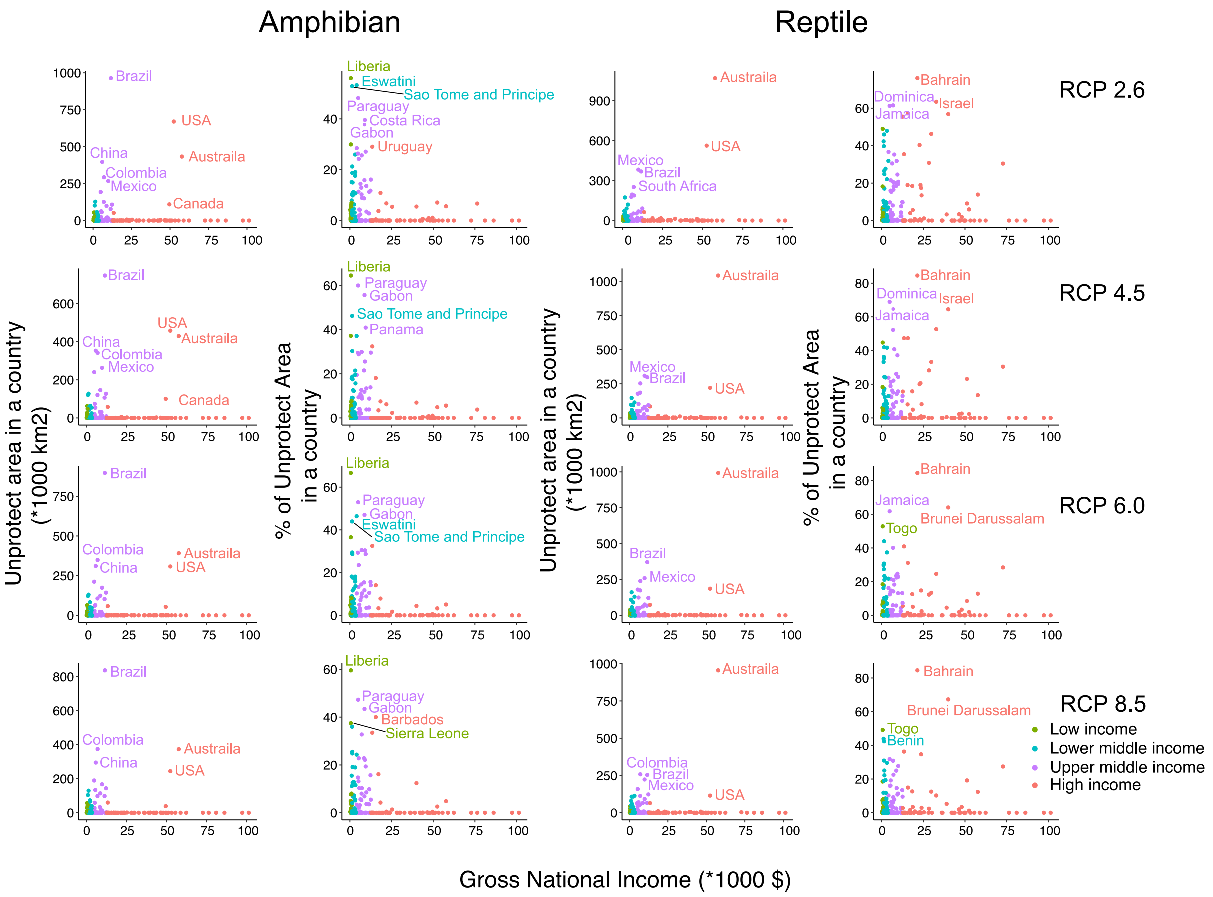

Supplementary Figure 25 Plot of unprotected area and gross national income at country level in future. Y-axis in the first and third columns represent the unprotected area in a country, in the second and fourth represent the percent of unprotected area occupy by the country's land area. Under RCP 4.5, the non-high-income countries account for 81.7% and 81.2% (unprotected area of a country > median unprotected area of all countries); the non-high-income countries account for 82.0% and 69.0% (Percent of unprotected area occupy the total land area of that country > 10%). Gross National Income (GNI) data is from https://data.worldbank.org/, we averaged GNI for each country from 2010-2014. Country class based on GNI: low income, GNI < \$1036; lower middle, \$1036 ≤ GNI < \$4045; upper middle, \$4045≤ GNI < \$12535; high income, GNI ≥ \$12535.

Supplementary Tables

Supplementary Table 1 Key studies evaluating global vertebrate diversity patterns and priority areas for conservation

Study	Resolution	Taxa	Location
Chen et al. 2017	0.1°*0.1°, 0.25°*0.25°, 0.5°*0.5°	Amphibians	China
Gallardo et al. 2017	1 km * 1 km	invasive terrestrial, freshwater, and marine species	Europe
Lehikoinen et al. 2018	100 km*100 km	Birds	Finland
Rodrigues et al. 2004	0.5° * 0.5°	Mammals, amphibians, freshwater turtles and tortoises, and birds	Global
Zhu et al. 2021	1 km * 1 km	amphibian, bird, Mammals, and reptiles	East of the USA
Araújo et al. 2011	16 km * 16 km	plants and terrestrial vertebrate	Europe
Hannah et al. 2007	1 km * 1 km, 1.8 km * 1.8 km, 50 km * 50 km	Birds, butterflies, mammals	Mexico, Cape Floristic,Western Europe
Hole et al. 2009	1° * 1°	Birds	sub-Saharan Africa

Thomas et al. 2012	10 km * 10 km	Bird and butterfly	Great Britain
Beale et al. 2013	0.5° * 0.5°	Birds	Tanzania

Supplementary Reference

Araújo, M. B., Alagador, D., Cabeza, M., Nogués-Bravo, D., & Thuiller, W. (2011). Climate change threatens European conservation areas. *Ecology Letters*, *14*(5), 484–492. https://doi.org/10.1111/j.1461-0248.2011.01610.x

Beale, C. M., Baker, N. E., Brewer, M. J., & Lennon, J. J. (2013). Protected area networks and savannah bird biodiversity in the face of climate change and land degradation. *Ecology Letters*, *16*(8), 1061–1068. https://doi.org/10.1111/ele.12139

Chen, Y., Zhang, J., Jiang, J., Nielsen, S., & He, F. (2017). Assessing the effectiveness of China's protected areas to conserve current and future amphibian diversity. *Diversity and Distributions*, *23*(2), 146–157. https://doi.org/10.1111/ddi.12508

Gallardo, B., Aldridge, D. C., González-Moreno, P., Pergl, J., Pizarro, M., Pyšek, P., Thuiller, W., Yesson, C., & Vilà, M. (2017). Protected areas offer refuge from invasive species spreading under climate change. *Global Change Biology*, *23*(12), 5331–5343. https://doi.org/10.1111/gcb.13798

Hannah, L., Midgley, G., Andelman, S., Araújo, M., Hughes, G., Martinez-Meyer, E., Pearson, R., & Williams, P. (2007). Protected area needs in a changing climate. *Frontiers in Ecology and the Environment*, *5*(3), 131–138. https://doi.org/10.1890/1540-9295(2007)5[131:paniac]2.0.co;2

Hole, D. G., Willis, S. G., Pain, D. J., Fishpool, L. D., Butchart, S. H. M., Collingham, Y. C., Rahbek, C., & Huntley, B. (2009). Projected impacts of climate change on a continent-wide protected area network. *Ecology Letters*, *12*(5), 420–431.

Lehikoinen, P., Santangeli, A., Jaatinen, K., Rajasärkkä, A., & Lehikoinen, A. (2018). Protected areas act as a buffer against detrimental effects of climate change-Evidence from large-scale, long-term abundance data. *Global Change Biology*, *25*(1), 304–313. https://doi.org/10.1111/gcb.14461

Rodrigues, A. S. L., Akçakaya, H. R., Andelman, S. J., Bakarr, M. I., Boitani, L., Brooks, T. M., Chanson, J. S., Fishpool, L. D. C., Fonseca, G. A. B. D., Gaston, K. J., Hoffmann, M., Marquet, P. A., Pilgrim, J. D., Pressey, R. L., Schipper, J., Sechrest, W., Stuart, S. N., Underhill, L. G., Waller, R. W., … Yan, X. (2004). Global Gap Analysis: Priority Regions

for Expanding the Global Protected-Area Network. *BioScience*, *54*(12), 1092–1100. https://doi.org/10.1641/0006-3568(2004)054[1092:ggaprf]2.0.co;2

Thomas, C. D., Gillingham, P. K., Bradbury, R. B., Roy, D. B., Anderson, B. J., Baxter, J. M., Bourn, N. A. D., Crick, H. Q. P., Findon, R. A., Fox, R., Hodgson, J. A., Holt, A. R., Morecroft, M. D., O'Hanlon, N. J., Oliver, T. H., Pearce-Higgins, J. W., Procter, D. A., Thomas, J. A., Walker, K. J., … Hill, J. K. (2012). Protected areas facilitate species' range expansions. *Proceedings of the National Academy of Sciences*, *109*(35), 14063–14068. https://doi.org/10.1073/pnas.1210251109

Zhu, G., Papeş, M., Giam, X., Cho, S.-H., & Armsworth, P. R. (2021). Are protected areas well-sited to support species in the future in a major climate refuge and corridor in the United States? *Biological Conservation*, *255*, 108982. https://doi.org/10.1016/j.biocon.2021.108982

Supplementary Table 2 Top 5 Family and Genus with percent of species number that are not predicted to be in PAs at present and future (RCP 4.5) by 2070 under the no-dispersal scenario

Amphibian		Reptile	
Current	Future	Current	Future
Family			
Heleophrynidae (75.0%)	Heleophrynidae (75.0%)	Xenotyphlopidae (100.0%)	Trogonophidae (60.0%)
Rhinatrematidae (50.0%)	Chikilidae (50.0%)	Trogonophidae (60.0%)	Gerrhopilidae (50.0%)
Brevicipitidae (44.4%)	Brevicipitidae (38.9%)	Gerrhopilidae (50.0%)	Acrochordidae (33.3%)
Pyxicephalidae (35.3%)	Telmatobiidae (38.9%)	Bipedidae (33.3%)	Bipedidae (33.3%)
Leiopelmatidae (33.3%)	Ceratobatrachidae (30.6%)	Xenosauridae (30.0%)	Cordylidae (27.4%)
Genus			
Arthroleptella (100%)	Heleophryne (100%)	Chioninia (100%)	Chioninia (100%)
Heleophryne (100%)	Anhydrophryne (100%)	Agamodon (100%)	Agamodon (100%)
Callulops (100%)	Quilticohyla (100%)	Ailuronyx (100%)	Haemodracon (100%)
Lyciasalamandra (100%)	Hylophorbus (100%)	Haemodracon (100%)	Hemicordylus (100%)
Copiula (100%)	Capensibufo (100%)	Adelophis (100%)	Acutotyphlops (100%)

Note: the number in brackets represents the number of species relating to Family or Genus only distributed outside PAs.
