# Supplementary material for: Global Protected Areas as refuges for amphibians and reptiles under climate change
Source: Nat Commun. 2023 Mar 13;14:1389. doi: 10.1038/s41467-023-36987-y (PMC10011414; doi:10.1038/s41467-023-36987-y)
Supplement: Supplementary file 3 — Description of Additional Supplementary Files [file 41467_2023_36987_MOESM3_ESM.docx]

File Name: Supplementary Data 1
Description: The references list for occurrence records.

File Name: Supplementary Data 2
Description: Species distribution model accuracy, and threshold transforming habitat suitability map to binary map (Presence/Absence). Following are the descriptions for columns:

**Species**: species scientific name.

**Group**: species is amphibian or reptile.

**AUC**: model accuracy index of area under the receiver operating characteristic curve

**TSS**: model accuracy index of true skill statistic

**threshold:** the value of species habitat suitability maps were transformed to binary distribution maps (presence/absence) with the threshold that maximizes TSS

File Name: Supplementary Data 3
Description: Climate change impact data, protected area coverage data generated in this study for amphibians. Following are the descriptions for columns:

**Species**: species scientific name.

**curareaAll:** species current range size.

**rcp26areaAll**: species range size under RCP 2.6 scenario.

**rcp45areaAll**: species range size under RCP 4.5 scenario.

**rcp60areaAll**: species range size under RCP 6.0 scenario.

**rcp85areaAll**: species range size under RCP 8.5 scenario.

**class, order, family, genus**: species taxonomy.

**redlistCategory**: species IUCN threatened categories.

**Category**: species are Threatened or NoThreatened. Threatened classified as Near Threatened, Vulnerable, Endangered, Critically Endangered, and Extinct based on IUCN.

**Range**: Large or small range species, species range size larger or small than the median range size of all species

**curareaIn:** species current range size inside Protected Areas (PAs).

**rcp26areaIn**: species range size inside PAs under RCP 2.6 scenario.

**rcp45areaIn**: species range size inside PAs under RCP 4.5 scenario.

**rcp60areaIn**: species range size inside PAs under RCP 6.0 scenario.

**rcp85areaIn**: species range size inside PAs under RCP 8.5 scenario.

**curareaOut:** species current range size outside Protected Areas (PAs).

**rcp26areaOut**: species range size outside PAs under RCP 2.6 scenario.

**rcp45areaOut**: species range size outside PAs under RCP 4.5 scenario.

**rcp60areaOut**: species range size outside PAs under RCP 6.0 scenario.

**rcp85areaOut**: species range size outside PAs under RCP 8.5 scenario.

**Loss26In**: percent of species range size loss inside PAs under RCP 2.6 scenario.

**Loss45In**: percent of species range size loss inside PAs under RCP 4.5 scenario.

**Loss60In**: percent of species range size loss inside PAs under RCP 6.0 scenario.

**Loss85In**: percent of species range size loss inside PAs under RCP 8.5 scenario.

**Loss26Out**: percent of species range size loss outside PAs under RCP 2.6 scenario.

**Loss45Out**: percent of species range size loss outside PAs under RCP 4.5 scenario.

**Loss60Out**: percent of species range size loss outside PAs under RCP 6.0 scenario.

**Loss85Out**: percent of species range size loss outside PAs under RCP 8.5 scenario.

**curInPA:** percent of species current range size inside Protected Areas (PAs).

**rcp26InPA**: percent of species range size inside PAs under RCP 2.6 scenario.

**rcp45InPA**: percent of species range size inside PAs under RCP 4.5 scenario.

**rcp60InPA**: percent of species range size inside PAs under RCP 6.0 scenario.

**rcp85InPA**: percent of species range size inside PAs under RCP 8.5 scenario.

File Name: Supplementary Data 4
Description: Climate change impact data, protected area coverage data generated in this study for reptiles. The descriptions for columns are same with Supplementary Data 3.

File Name: Supplementary Data 5

Description: Species list are not in PAs at present and under climate change scenarios. Following are the descriptions for columns:

**Species**: species scientific name.

**curareaAll:** species current range size.

**rcp26areaAll**: species range size under RCP 2.6 scenario.

**rcp45areaAll**: species range size under RCP 4.5 scenario.

**rcp60areaAll**: species range size under RCP 6.0 scenario.

**rcp85areaAll**: species range size under RCP 8.5 scenario.

**class, order, family, genus**: species taxonomy.

**curareaIn:** species current range size inside Protected Areas (PAs).

**rcp26areaIn**: species range size inside PAs under RCP 2.6 scenario.

**rcp45areaIn**: species range size inside PAs under RCP 4.5 scenario.

**rcp60areaIn**: species range size inside PAs under RCP 6.0 scenario.

**rcp85areaIn**: species range size inside PAs under RCP 8.5 scenario.

**redlistCategory**: species IUCN threatened categories.

**Category**: species are Threatened or NoThreatened. Threatened classified as Near Threatened, Vulnerable, Endangered, Critically Endangered, and Extinct based on IUCN.

**Range**: Large or small range species, species range size larger or small than the median range size of all species

File Name: Supplementary Data 6

Description: Conservation gaps for global amphibians and reptiles and gross national income at country level. Following are the descriptions for columns:

**COUNTRY:** country or region name.

**ISO and Code**: abbreviation of country name.

**Continent**: the continent of country belongs to.

**Area**: country range.

**AmpGap26**: amphibian conservation conservation gap areas in a country under RCP 2.6 scenario.

**AmpGap45**: amphibian conservation gap areas in a country under RCP 4.5 scenario.

**AmpGap60**: amphibian conservation gap areas in a country under RCP 6.0 scenario.

**AmpGap85**: amphibian conservation gap areas in a country under RCP 8.5 scenario.

**RepGap26**: reptile conservation gap areas in a country under RCP 2.6 scenario.

**RepGap45**: reptile conservation gap areas in a country under RCP 4.5 scenario.

**RepGap60**: reptile conservation gap areas in a country under RCP 6.0 scenario.

**RepGap85**: reptile conservation gap areas in a country under RCP 8.5 scenario.

**AmpGap26Per**: percent of amphibian conservation gap areas in a country under RCP 2.6 scenario.

**AmpGap45Per**: percent of amphibian conservation gap areas in a country under RCP 4.5 scenario.

**AmpGap60Per**: percent of amphibian conservation gap areas in a country under RCP 6.0 scenario.

**AmpGap85Per**: percent of amphibian conservation gap areas in a country under RCP 8.5 scenario.

**RepGap26Per**: percent of reptile conservation gap areas in a country under RCP 2.6 scenario.

**RepGap45Per**: percent of reptile conservation gap areas in a country under RCP 4.5 scenario.

**RepGap60Per**: percent of reptile conservation gap areas in a country under RCP 6.0 scenario.

**RepGap85Per**: percent of reptile conservation gap areas in a country under RCP 8.5 scenario.

**GNI**: gross national income at country level.

**incomeGroup**: country groups. country class based on GNI: low income, GNI < $1036; lower middle, $1036 ≤ GNI < $4045; upper middle, $4045≤ GNI < $12535; high income, GNI ≥ $12535.

File Name: Supplementary Data 7

Description: The species with occurrence records are not included in upload occurrence datasets. Following are the descriptions for columns:

**ScientificName:** species scientific name.

**Region**: The location that species is found.

**Contacts**: Person who have the records of related species.

**Email**: The email address of contacts.
